# Binding of SARS-CoV Covalent Non-Covalent Inhibitors to the SARS-CoV-2 Papain-Like Protease and Ovarian Tumor Domain Deubiquitinases

**DOI:** 10.3390/biom11060802

**Published:** 2021-05-28

**Authors:** Dakshinamurthy Sivakumar, Matthias Stein

**Affiliations:** Molecular Simulations and Design Group, Max Planck Institute for Dynamics of Complex Technical Systems, Sandtorstrasse 1, 39106 Magdeburg, Germany; sivakumar@mpi-magdeburg.mpg.de

**Keywords:** deubiquitinase, OTUB2, papain-like protease, SARS-CoV-2, drug design, molecular dynamics, covalent docking

## Abstract

The urgent need for novel and effective drugs against the SARS-CoV-2 coronavirus pandemic has stimulated research worldwide. The Papain-like protease (PLpro), which is essential for viral replication, shares a similar active site structural architecture to other cysteine proteases. Here, we have used representatives of the Ovarian Tumor Domain deubiquitinase family OTUB1 and OTUB2 along with the PLpro of SARS-CoV-2 to validate and rationalize the binding of inhibitors from previous SARS-CoV candidate compounds. By forming a new chemical bond with the cysteine residue of the catalytic triad, covalent inhibitors irreversibly suppress the protein’s activity. Modeling covalent inhibitor binding requires detailed knowledge about the compounds’ reactivities and binding. Molecular Dynamics refinement simulations of top poses reveal detailed ligand-protein interactions and show their stability over time. The recently discovered selective OTUB2 covalent inhibitors were used to establish and validate the computational protocol. Structural parameters and ligand dynamics are in excellent agreement with the ligand-bound OTUB2 crystal structures. For SARS-CoV-2 PLpro, recent covalent peptidomimetic inhibitors were simulated and reveal that the ligand-protein interaction is very dynamic. The covalent and non-covalent docking plus subsequent MD refinement of known SARS-CoV inhibitors into DUBs and the SARS-CoV-2 PLpro point out a possible approach to target the PLpro cysteine protease from SARS-CoV-2. The results show that such an approach gives insight into ligand-protein interactions, their dynamic character, and indicates a path for selective ligand design.

## 1. Introduction

The most recent pandemic, the SARS-CoV-2 coronavirus outbreak, is one of the largest threats to the human population worldwide and still significantly increasing in absolute numbers. According to WHO reports, it is one of the worst pandemics seen during recent times. The SARS-CoV-2 genome encodes for 29 proteins, of which 4 are structurally encoding and make up the virus’s actual structure. Thus far, the spike (S) protein and the main protease MLpro have received the most attention in drug design. The X-ray crystal structure of the six-helical bundle (6-HB) core of the HR1 and HR2 domains in the SARS-CoV-2 spike protein S2 subunit was released [1]. The non-structured proteins (Nsp’s 1–16) are obtained after cleavage of a large polyprotein into 16 smaller proteins in which the main protease (Mpro) plays a significant role.

One of the best-characterized drug targets among coronaviruses is the main protease (Mpro, also called 3CLpro), for which the crystal structure was solved at low temperature [2] and room temperature [3]. Peptidomimetic α-ketoamides were shown to bind in the substrate-binding cleft located between domains I and II of the Mpro. Virtual screening studies have also explored this protein as a potential target against Covid-19 [4]. They revealed known antiviral compounds such as velpatasvir and ledipasvir as drug candidates. The Papain-like protease (PLpro), an enzyme essential for processing the polyproteins translated from the viral RNA, is less explored as a drug target.

Similar to that Severe Acute Respiratory Syndrome coronavirus (SARS-CoV) [5], the Papain-like protease (PLpro) of SARS-CoV-2 can be a potential target for antiviral drug development since it is essential for generating a functional replication complex (see above). The PLpro protease from SARS-CoV is structurally similar to the deubiquitinase (DUB) USP7 and shows deubiqutinating activity [6]. The triad of catalytic site residues Cys/His/Asp is essential for its DUB activity and conserved in the above PLpro enzymes. Cysteine proteases react with a variety of electrophilic or ‘warhead’ functional groups from covalent inhibitor molecules. These warhead inhibitors typically function by first forming a non-covalent pre-complex within the cysteine protease active site. The warhead group of the inhibitor is positioned in proximity of the reactive cysteine nucleophile. The X-ray structure of the catalytic domain of the Nsp3 SARS-CoV PLpro showed that, like in some other cysteine proteases, SARS-CoV PLpro could react with electrophilic warheads and incorporate an inhibitor from a nucleophilic attack of the cysteine thiolate [7]. For SARS-CoV, there are several designed covalent inhibitors in the literature [8].

The current state of drug development and medicinal chemistry efforts towards SARS-CoV-2 and Covid-19 treatments are summarized in reference [9]. With the advancement of computational power, virtual high-throughput screening of millions of compounds can be performed in a relatively short time [10]. Smith and Smith, for example, have investigated the re-purposing of existing drug molecules to bind to an MD ensemble of the SARS-CoV-2 spike protein at its host receptor region or its interface with human ACE2 [10].

There are a plethora of computational approaches tackling selected protein targets from SARS-CoV-2. They range from virtual screening of different ligand databases or selective subsets to specific protein targets, neural networks, machine learning, and artificial intelligence.

A recent study shows that the screening of the large ZINC database entries plus an in-house database with antiviral compounds against all SARS-CoV-2 proteins gave hits such as on-the-market antiviral drugs (ribavirin, valganciclovir, and thymidine), antibiotics (cefpiramide, sulfasalazine, phenethicillin, lymecycline, demeclocycline, doxycycline, oxytetracycline, and tigecycline), and anti-asthmatic drugs (montelukast, fenoterol, and reproterol). It remains to be demonstrated how reliable and trustworthy these suggested compounds will be [11]. The Papain-like protease PLpro was chosen as the target in computational studies focusing on existing drug re-purposing and a conventional ligand docking into the S3/S4 pockets of the enzyme’s active site. As a result, 16 already FDA approved drugs, including the now discarded malaria drug chloroquine and the asthma drug formoterol, were suggested [12].

Since PLpro is also functional in removing post-translational signaling tags like ubiquitin and interferon-stimulated gene product 15 (ISG15), it dampens inflammation response and antiviral signaling [13]. Drugs that target SARS-CoV-2 PLpro may also be effective as treatments or protection against Covid-19 by reducing the viral load and reinstating an innate immune response [14]. Given this conceptual and functional similar role to human deubiquitinases (DUBs), we take a physiologically and chemically motivated approach instead of high-throughput screening.

Otubain-1 and Otubain-2 (also termed OTUB1 and OTUB2) are cysteine proteases like PLpro with the same Cys/His/Asp, Asn catalytic residue triad [15]. They are members of the DUB superfamily with ubiquitin-cleavage activity like PLpro and representatives of the ovarian tumor domain (OTUs) subfamily. Only very recently, a screening of an electrophilic fragment library revealed the first selective Otubain-2 inhibitors [16]. These inhibitors also use a chemical warhead to establish a covalent cysteinate protein-ligand complex, as reported for the SARS-CoV PLpro (see above). Since covalent inhibition was previously reported for SARS-CoV, it also may appear feasible for SARS-CoV-2.

Modeling the binding of covalently bound ligands is a multi-step process and requires more computational time than standard ligand virtual screening. Since the binding pocket has to accommodate the pre-reactive ligand, the relative orientation of nucleophile (here cysteinate) and electrophile must be taken into account. When the binding site accessibility of the target cysteine is initially increased, the result from covalent docking is more accurate. It shows improved binding mode predictions (RMSD) and significantly lower computational expense than mere covalent docking [17].

We assess and validate the quality of modeling the covalent OTUB2 inhibitors by refining their co-crystallized structures using molecular dynamic (MD) simulations. By monitoring ligand fluctuations, structural re-orientation, and deviations from the crystal structure, stringent and covalent cysteinate-ligand binder modeling protocols are established. It may be challenging to develop PLpro inhibitors specific for the viral protease without blocking cellular human DUBs. Thus, we perform covalent docking for compounds **3**, **4**, and **5** and non-covalent docking for **1**, **2**, and **6** plus MD refinement of a representative set of known SARS-CoV inhibitors into OTUB2, OTUB1, and the PLpro from SARS-CoV-2 to probe their inhibitor binding and rationalize a DUB selectivity. We point out that the structural differences in cellular DUBs suggest that these enzymes may be different enough to be selectively targeted.

## 2. Results and Discussion

### 2.1. Covalent OTUB2 Inhibitors

The Ovarian tumor domain (OTU) deubiquitinylases show a DUB activity and a functional similarity to the SARS-CoV-2-PLpro. Thus, our computational approach was validated and benchmarked by modeling the recently discovered covalent inhibitors of OTUB2 [16]. The screening of a library of mild electrophile fragments as pre-reactive compounds revealed irreversible chemical bonds with the catalytic Cys51 residue. A total of 11 reported selective covalent inhibitors for OTUB2 were co-crystallized by high throughput crystallization (PDB entries from 5QIP to 5QIZ (Table S1)) and selected for MD refinement studies to validate our molecular dynamics protocol. We assessed the applicability and accuracy of the OPLS2005 force field plus the correct structural reproduction of the covalent protein-ligand cysteine-carbon bond. Given the short timescale of multiple MD runs, the focus was on the ligands’ conformational re-orientation and rotational flexibility rather than protein conformational changes. From MD refinement, steric clashes were removed, and the dynamic nature of ligand-protein interactions became apparent. 

For each of the 11 OTUB2-ligand complexes, we carried out short MD refinement simulations of 3 × 100 ns. The trajectories were analyzed to rationalize the stability of the covalent ligand-protein complex and identify crucial interactions. Table S1 shows the co-crystallized ligand-OTUB2 structures and the MD refined structures.

#### 2.1.1. OTUB2 (5QIP) in Complex with PCM-0102153

Simulations of OTUB2 with the compound PCM-0102153 (benzyl acetylcarbamate; PDB id: 5QIP) showed that the covalent inhibitor-cysteine bond was stable throughout the simulation (distance 1.81 ± 0.04 Å vs. 1.77 Å in the crystal structure). The ligand remained in a conformation close to its initial position (with ligand RMSD of 1.23 ± 0.3 Å) during the first half of the simulation (Table S1). The benzyl ring underwent a rotational motion in the second half of the simulation time (to be seen from ligand RMSF and ligand torsional profile). Hydrogen bonding interactions with protein residues Cys51, Arg49, and Ser223 were persistent for more than 85% of the simulation time. Asp48 shows interaction with the compound for nearly 50% of the simulation time (see Figure S1).

#### 2.1.2. OTUB2 (5QIQ) in Complex with PCM-0103050

The OTUB2-PCM-0103050 (*N*-[(4-bromothiophen-2-yl)-methyl] acetamide) complex (PDB id: 5QIQ) showed a flexible rotational motion of the thiophene ring. Here, interactions with Cys51, Arg49, and Ser223 were most relevant and showed strong and stable hydrogen bond interactions with the ligand atoms (Figure S2); the ligand RMSD was around 1 Å (Table S1). 

#### 2.1.3. OTUB2 (5QIR) in Complex with PCM-0102305

The third complex (PDB id: 5QIR) with OTUB2 in complex PCM-0102305 (*N*-[(4-fluoro-3-methylphenyl)-methyl] acetamide) showed that the ligand orientation was very stable throughout the simulation. In this case, the Arg49 and Ser223 residues showed stable and persistent hydrogen bond interactions with ligand atoms for more than 95% of the simulation time. Asp48 and Cys51 showed interactions for more than 50% and 30% of the simulation time (Figure S3), and, in this case, the ligand RMSD was around 1 Å (Table S1).

#### 2.1.4. OTUB2 (5QIS) in Complex with PCM-0102500

MD results of PCM-0102500 (*N*-(5-methyl-1,2-oxazol-3-yl) acetamide) in complex with OTUB2 (PDB id: 5QIS) showed that Arg49 and Ser223 formed strong hydrogen bond interactions with ligand atoms for more than 90% of the simulation time on average. Cys51 and Asp48 showed interactions for nearly 80% of the simulation time on average (Figure S4), and the ligand RMSD is just 0.35 Å (Table S1).

#### 2.1.5. OTUB2 (5QIT) in Complex with PCM-0102821

The fifth complex (PDB id: 5QIT) with the compound PCM-0102821 (*N*-[(E)-(3-methylphenyl) methylidene] acetamide) displayed a rotation of the solvent-exposed phenyl ring. In this complex, we can identify that Arg49 and Ser223 formed hydrogen bond interactions with ligand atoms for more than 95% of the simulation time with a ligand RMSD of around 1 Å (Table S1). Cys51 and Asp48 showed interactions for nearly 35% of the simulation time (Figure S5).

#### 2.1.6. OTUB2 (5QIU) in Complex with PCM-0103011

The compound PCM-0103011 (*N*-3-[3-(trifluoromethyl) phenyl] prop-2-yn-1-ylacetamide) in complex with OTUB2 (PDB id: 5QIU) showed a higher degree of rotational flexibility of the terminal phenyl ring due to the methyl group. Still, the ligand RMSD was around 1.1 Å (Table S1). Arg49, Ser223 showed strong hydrogen bond interaction for more than 95% of the simulation time. For less than 10% of the simulation time, some hydrophobic interactions were also observed by active site residues His224 and Lys221 (Figure S6).

#### 2.1.7. OTUB2 (5QIV) in Complex with PCM-0102998

OTUB2 in complex with the compound PCM-0102998 (*N*’-acetyl-2-[(3R)-1,1-dioxo-1 lambda~6~-thiolan-3-yl] acetohydrazide) (PDB id: 5QIV) showed a rigid ligand conformation throughout the simulation. Strong hydrogen bond contacts were detected between Arg49, Cys51, Ser223, and the ligand for nearly 90% of the simulation time with a ligand RMSD of 0.8 Å (Table S1). Glu174 shows a water-mediated hydrogen bond interaction for 30% of the simulation time (Figure S7).

#### 2.1.8. OTUB2 (5QIW) in Complex with PCM-0102660

The covalent OTUB2 complex (PDB id: 5QIW) with compound PCM-0102660 (*N*-[(E)-(4-methylphenyl) methylidene] acetamide) shows that the ligand was rotationally flexible throughout the simulation due to its extra methyl group between the amide bond and the terminal phenyl ring; still the ligand RMSD is only 0.7 Å (Table S1). Strong hydrogen bond contacts between Arg49, Ser223 were persistent for nearly 98% of the simulation time. Peptide bond NH groups of Cys51 and Asp48 are hydrogen bond donors to the amide carbonyl oxygen atom for 60% and 35% of the simulation time, respectively (Figure S8). 

#### 2.1.9. OTUB2 (5QIX) in Complex with PCM-0103007

The covalent OTUB2-inhibitor complex (PDB id: 5QIX) with PCM-0103007 (*N*-(3-phenylprop-2-yn-1-yl) acetamide) possessed a rotatable terminal phenyl ring due to its flexible propyl link to the acetamide. Arg49, Cys51, and Ser223 showed strong interactions with the ligand for 99%, 78%, and 88% of the simulation time, respectively (Figure S9), but with the ligand RMSD of 0.4 Å (Table S1). 

#### 2.1.10. OTUB2 (5QIY) in Complex with PCM-0102954

Simulation of OTUB2 complex (PDB id: 5QIY) with the compound PCM-0102954 (*N*’-acetyl-2-chlorobenzohydrazide) showed that the ligand was rotationally flexible and had a low ligand RMSD of 0.6 Å (Table S1). The residues Arg49, Glu174 and Ser223, showed strong hydrogen bonds for nearly 60% of the simulation time. The cysteine, however, did not form hydrogen-bonding interactions with the ligand (Figure S10). 

#### 2.1.11. OTUB2 (5QIZ) in Complex with PCM-0103080

The last complex (PDB entry 5QIZ) of OTUB2 with the compound PCM-0103080 (*N*-[(5-chlorothiophen-2-yl) methyl] acetamide) showed a very stable interaction between the protein and ligand throughout the simulation with the ligand RMSD of 1.3 Å (Table S1). A pi-pi stacking between the thiophene ring of the ligand with His224 of the protein was contributing. The long-living interactions with the peptide NH groups of Cys51 and Arg49 were present during 95% of the simulation time. Ser223 was a hydrogen bond acceptor of the amide bond NH for nearly 80% of the simulation time (Figure S11).

Overall, the refinement of the covalent OTUB2 inhibitors gave structural parameters such as ligand RMSD and CysS51-C_ligand_ distances in perfect agreement with the co-crystallized structures with an average of 1.7 Å and 1.8 Å, respectively (Table S1).

### 2.2. Binding of Known Covalent SARS-CoV PLpro Inhibitors to OTUB2/OTUB1/SARS-CoV-2 PLpro

Covalent cysteine protease inhibition proceeds via a nucleophilic attack of the thiolate on the electrophilic carbon of the warhead group, then forming a covalently modified enzyme–inhibitor complex [18]. Examples of such reactive warhead groups known to inhibit cysteine proteases include aldehydes, epoxy-ketones, Michael acceptors, activated ketones, activated esters, vinyl sulfones, acrylamides, alkynes, alkyl halides, and nitriles [19,20,21].

**Figure 1:**
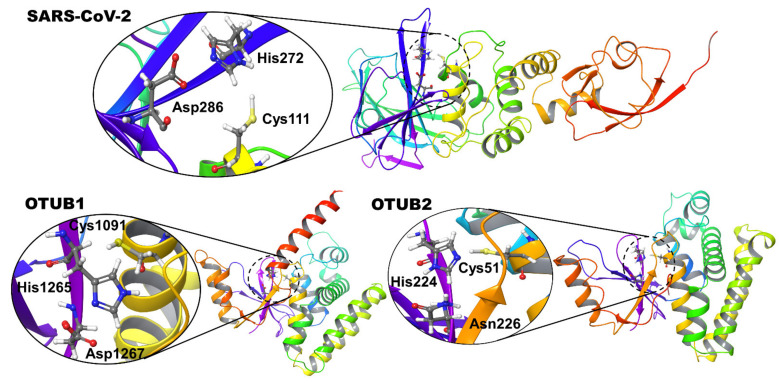
Structural details of the deubiquitinase-like arrangement of active site residues of SARS-CoV-2 PLpro and OTUs. The catalytic triads made up of Cys-His-Asp/Asn residues are shown as insets.

In preparation for normal docking and covalent docking, the co-crystallized ligands in SARS CoV-2 (PDB id: 6WUU) and OTUB2 (PDB id: 5QIY) were removed. For OTUB1, the PDB entry 2ZFY was used. Both refer to apoprotein structures in the absence of ubiquitin. The active sites of these OTU deubiquitinases show the conserved catalytic triad, which is also present in the PLpro (Cys-His-Asp/Asn) (Figure 1).

**Figure 2:**
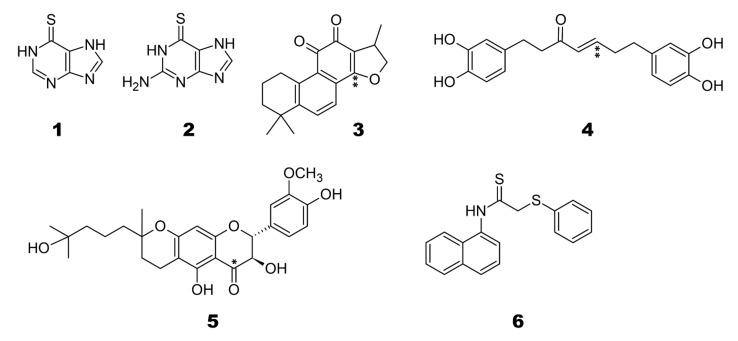
Known SARS-CoV PLpro inhibitors used as candidates during covalent and non-covalent ligand docking against OTUB1/OTUB2 and SARS-CoV-2 PLpro. Studied covalent linkage sites for ‘nucleophilic addition to a double bond’ marked (*) and site which follows ‘Michael addition’ marked (**) in the compounds.

Baez-Santos et al. reviewed the SARS-coronavirus papain-like proteases PLpro and 3CLpro as targets for the design of antiviral drug molecules against SARS-CoV and MERS [8]. We refer to a sub-selection as representatives of some classes of compounds. Here, we assess the binding poses and possible selectivity of known SARS-CoV cysteine protease inhibitors. The selected subset of SARS-CoV PLpro inhibitors for the binding PLpro of SARS-CoV-2, Otub1, and Otub2 is given in Figure 2.

These are representatives of the classes of thiopurine inhibitors compounds **1** [22] and **2** [23], tanshinones **3** [24], diarylheptanoids **4** [25], geranylated flavonoids compound **5** [26], and compound **6** from yeast-based screening [27].

**Figure 3:**
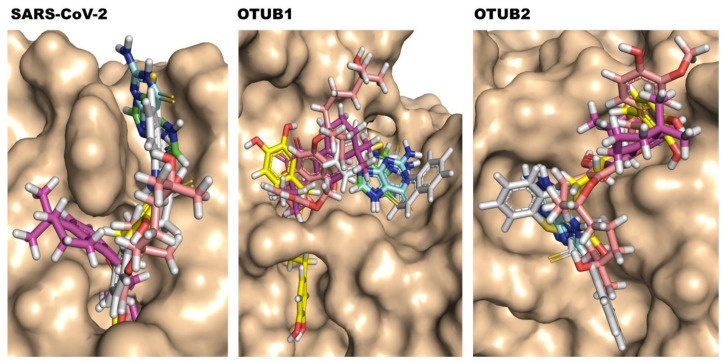
Top binding pose of selected SARS-CoV PLpro inhibitors docked into SARS-CoV-2 PLpro, OTUB1, and OTUB2 active sites. The protein is shown as surface and inhibitors are shown in the sticks with different colors (carbon atom) to differentiate them. Compound **1** in green, Compound **2** in aquamarine, Compound **3** in magenta, Compound **4** in yellow, Compound **5** in pink, and Compound **6** in grey.

Compounds **1**, **2** are clearly non-covalent inhibitors (also observed by [23]), and also Compound **6** has no site for covalent cysteine binding. Thus, we carried out non-covalent docking using Glide XP with standard parameters (refer to method section for details). The Compounds **3** and **4**, however, which belong to α,β-unsaturated carbonyl compounds class, are tentative covalent inhibitors and prone to 1,4 addition and 1,2 additions, with the first preferred. Compounds **3** and **4** are molecules which undergo Michael additions based on previous reports [23]. Only in the case of Compound **5**, “nucleophilic addition to a double bond” reaction mechanism was preferable, and covalent docking with the catalytic cysteine residue and the thiocarbonyl group of the compound was performed (see Figure 2). Table 1 gives the hydrogen bond interactions and pi-pi interactions detected from non-covalent and covalent docking, and relevant active site residues are highlighted. The superimposed docked top conformations of all the compounds **1** to **6** with the respective targets are shown in Figure 3.

### 2.3. Molecular Dynamics Refinement of SARS-CoV-2 PLpro Inhibitors

**Figure 4:**
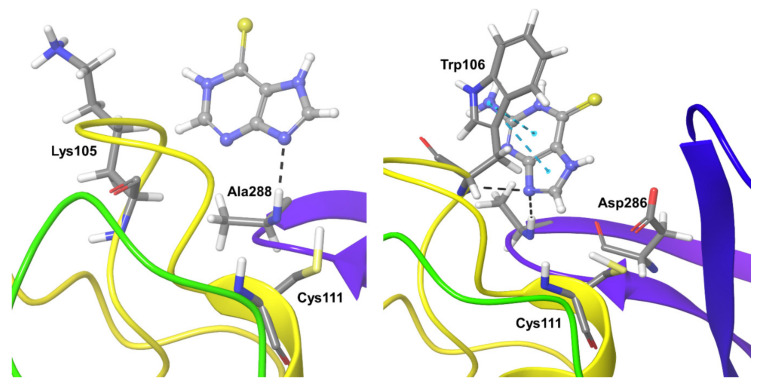
Persistent protein-ligand interactions during MD simulations of Compound **1** (**left**) and **2** (**right**) with SARS-CoV-2 Plpro. The compounds are shown in ball and stick representation, the strong and moderately interacting residues represented in thick tubes, and weak interactions (less than 30%) represented in thin tubes.

Compound **1** formed hydrogen bond interactions with Lys105 for 50% of the simulation time. During the remaining 30% of the simulation time, it formed various types of interactions such as hydrophobic interactions, electrostatic interactions, and water-mediated interactions. Ala288 also showed direct and water-mediated hydrogen bond interactions for nearly 70% of the simulation time (Figure 4). Other short-lived ionic and hydrogen bonding interactions were observed with Lys94, Pro96, Gln97, and Trp106.

Compound **2** showed persistent hydrogen bonding interactions with Ala288 for nearly 80% of the simulation time, and Trp106 showed hydrogen bonding and hydrophobic interactions for almost 60% of the simulation time. Asp286 showed water-mediated hydrogen bond interactions for nearly 40% of the simulation time (Figure 4).

**Figure 5:**
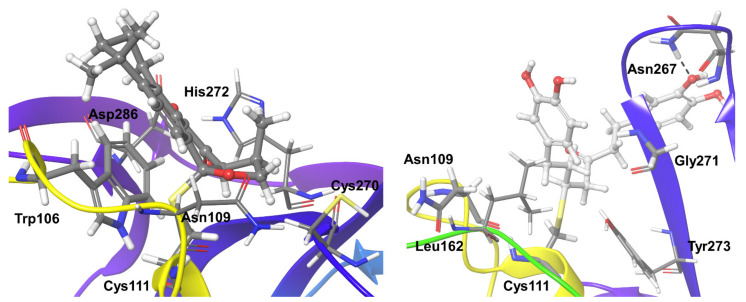
Stable interactions observed during MD simulations of Compound **3** (**left**) and **4** (**right**) with SARS-CoV-2 Plpro. The compounds are shown in ball and stick representation, stable and moderately stable interacting protein residues are given as thick tubes, and weak interactions (for less than 30%) are represented as thin tubes.

Compound **3**, which follows Michael addition reaction with the SARS-CoV-2 PLpro, formed stable hydrophobic interactions for nearly 50% of the simulation and additional hydrogen bond interactions with the covalently bound residue Cys111. Trp106 also forms hydrophobic interactions with Compound **3** (Figure 5). Few water bridges are also observed between the compound with residues Asn109, Cys270, His272, and Asp286 for less than 10% of the simulation time. Based on the RMSD analysis of the ligand and protein, it is clear that there are no large conformational changes for the ligand during the initial two-thirds of the simulation time. Larger fluctuations can be observed during the longer simulations, and also the protein binding region is flexible.

Compound **4** also followed Michael addition and formed a covalent interaction with active site Cys111, plus stable hydrophobic interactions with the same residue during the entire simulation time. Several mixed interactions like direct hydrogen bonding and water-mediated hydrogen bond interactions were observed during nearly 30–40% of the simulation time with Asn109, Asn267, Gly271. In addition, residues Leu162 and Tyr273 formed hydrophobic and water-mediated interactions with Compound **4** (Figure 5).

**Figure 6:**
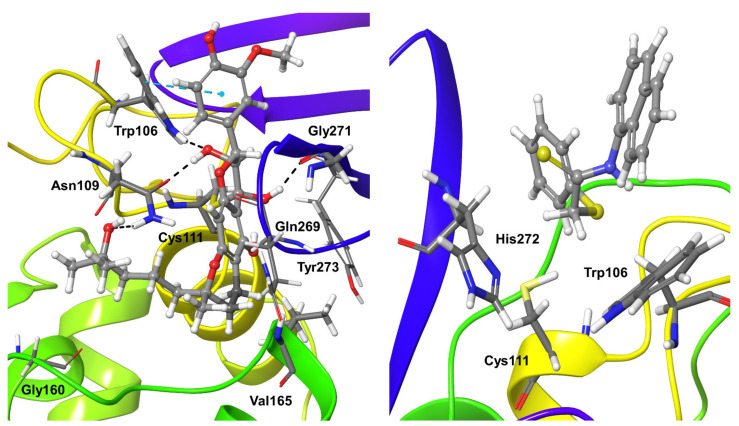
Stable interactions observed during MD refinement of top binding poses of compound **5** (**left**) and **6** (**right**) with SARS-CoV-2 Plpro. The inhibitors are shown in ball and stick representation, stable and moderately stable ligand-protein interactions are represented as thick tubes, short time interactions (less than 30%) are represented as thin tubes.

Compound **5** displayed stable interactions with the protein for almost two-thirds of the simulation time, and only minor fluctuations were observed during the rest of the trajectory. The compound binding was stabilized by a larger number of hydrogen bonding interactions plus water-mediated and hydrophobic interactions. PLpro residues Asn109 and Gly271 formed stable hydrogen bond interactions for more than 90% and 80% of the simulation time. Trp106 formed hydrogen bond interactions for 60% of the simulation time and also weaker hydrophobic interactions. Val165 formed water-mediated hydrogen bond interactions with Compound **5** for around 60% of the simulation time. Short-living water-mediated interaction could be observed for less than 20% of trajectories with residues Gly160, Gln269, and Tyr273 (Figure 6).

Compound **6** binding was dominated by weak and short-living hydrophobic interactions with residues Trp106, His272, Leu290, and Lys292 for only 8 to 14% of the simulation time (Figure 6). Residues Ala288 and Leu290 exhibited unstable hydrogen bonding interactions for only 5% of the simulation time. Likewise, hydrophobic interactions of the ligand with residues Val20, Pro96, and Leu289 were short in time and occurred during 3–5% of the simulation time.

### 2.4. Molecular Dynamics Refinement of OTUB1-Bound Inhibitors

**Figure 7:**
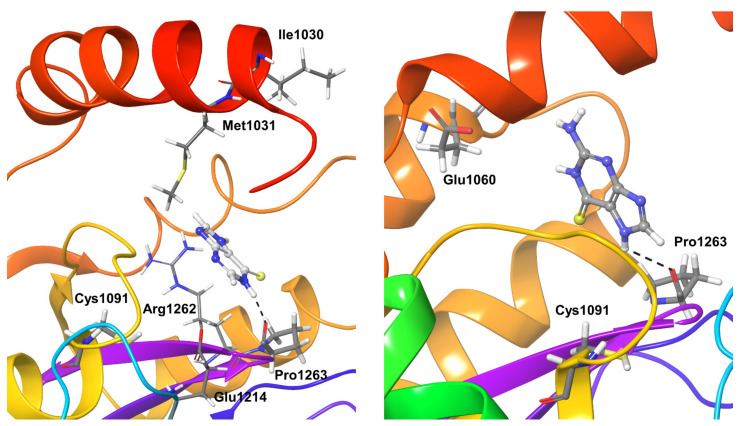
Stable interactions observed during MD refinement simulations of top-binding poses of Compound **1** (**left**) and **2** (**right**) with OTUB1. The inhibitors are shown in ball and stick representation, stable and moderately stable protein-ligand interactions are represented as thick tubes, and short-time interactions (for less than 30%) are represented as thin tubes.

Compound **1** binding to OTUB1 was similar to that in SARS-CoV-2. Protein residue Pro1263, which only forms hydrogen bond interactions (direct and water-mediated), was detectable for just 40% of the simulation time. Other less significant interactions were found for nearly 20–40% of the simulation time with the OTUB1 residues Ile1030, Met1031, Glu1214, and Arg1262 (Figure 7). Compound **2** showed significant interactions with Pro1263 and Glu1060 during nearly 80% of the simulation time; no other long-living interactions with the protein could be characterized (Figure 7).

**Figure 8:**
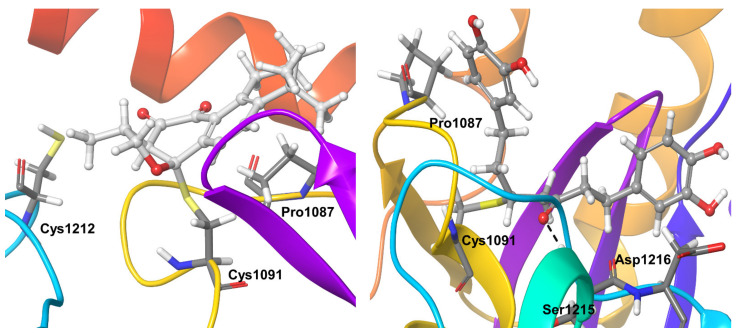
Stable inhibitor-protein interactions were observed during MD refinement simulations of top poses of Compound **3** (**left**) and **4** (**right**) with OTUB1. The inhibitors are shown in ball and stick representation, stable and moderately stable ligand-protein interactions are given as thick tubes, and flexible interactions (during less than 30%) are shown as thin tubes.

Compound **3**, covalently bound to residue Cys1091, showed very stable hydrophobic interactions throughout the simulations. OTUB1 residue Pro1087 showed hydrophobic interactions for nearly 40% of the simulation and water-mediated hydrogen bond interaction for another 20% of the simulation time. Cys1212 also showed hydrogen bond interactions during almost 30% of the simulation time (Figure 8).

Compound **4** showed hydrogen bonding interactions with residue Ser1215 for almost the entire simulation time. The covalently bound active site residue Cys1091 showed stable hydrophobic interactions during the entire simulation. OTUB1 residues Pro1087 and Asp1216 were additional protein residues that showed interactions for nearly 40% of the simulation time. Pro1087 showed hydrophobic and water-mediated hydrogen bond interactions, Asp1216 showed water-mediated and direct hydrogen bond interactions with the inhibitor (Figure 8).

**Figure 9:**
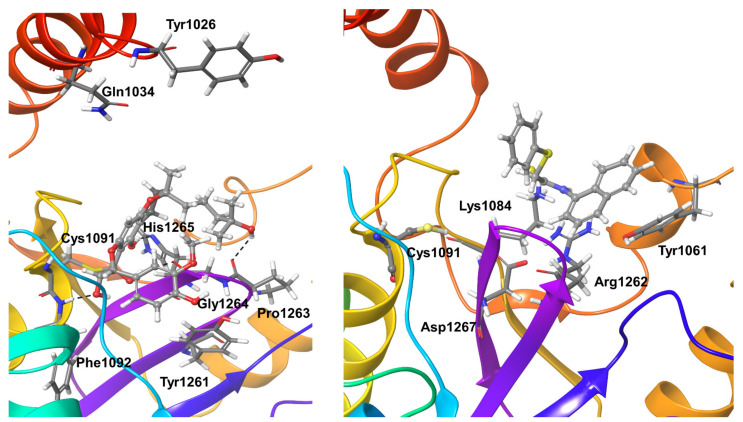
Stable interactions during MD refinement simulations of top poses for Compound **5** (**left**) and **6** (**right**) with OTUB1. The compounds are shown in ball and stick representation, stable and moderately stable ligand-protein interactions are given as thick tubes, and short-living interactions (preset for less than 30%) are represented as thin tubes.

Compound **5** showed stable hydrogen bond interactions for more than 95% of the simulation time with OTUB1 residues Phe1092 and Gly1264. His1265 shows hydrogen bond interactions for around 85% of the simulation time. Tyr1026, Gln1034, Tyr1261, and Pro1263 undergo interactions for at least 40% of the simulation time. The above four residues, except Tyr1261 (which showed water-mediated hydrogen bond interactions), showed direct hydrogen bond interactions with the compound. As expected, the active site residues and the covalently-fixed Cys1091 showed stable short-range hydrophobic interactions with the inhibitor compound (Figure 9).

Compound **6** shows two strong water-mediated interactions for nearly 75% of the simulation time with OTUB1 residues Tyr1084 and Asp1267. Residues Tyr1061 and Arg1262 show hydrophobic interactions for nearly 40% of the simulation time (Figure 9).

### 2.5. Molecular Dynamics Refinement of OTUB-2 Inhibitors

**Figure 10:**
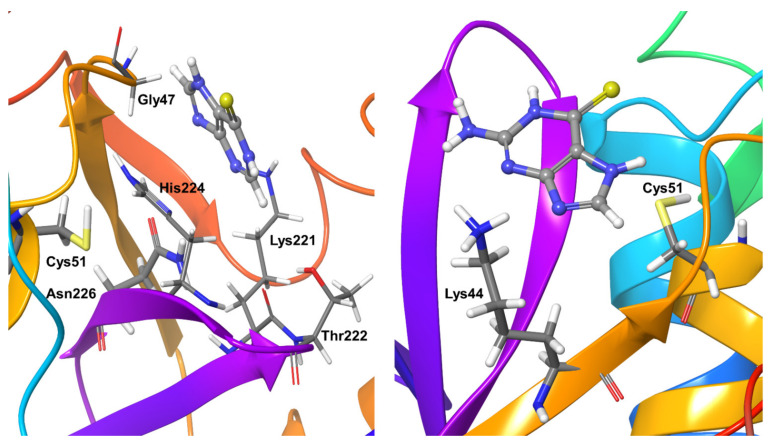
Stable protein-ligand interactions during MD refinement simulations for Compound **1** (**left**) and **2** (**right**) with OTUB-2. The inhibitors are shown in ball and stick representation, stable and moderately interaction with protein residues are shown as thick tubes, short-term interactions (present for less than 30% of simulation time) are shown as thin tubes.

Compound **1** showed hydrogen bonding interactions for nearly half of the simulation time with the active site residue Asn226 and long-living hydrophobic interactions with another active site residue, i.e., His224. OTUB2 residues Gly47, Lys221, and Thr222 showed hydrogen bonding interactions with the ligand for nearly 20–40% of the simulation time (Figure 10). There was no additional stable (present for >65% of simulation time) protein-ligand interaction. As for hydrophobic interactions, Compound **2** only comes close to Lys44 throughout the simulation time (Figure 10).

**Figure 11:**
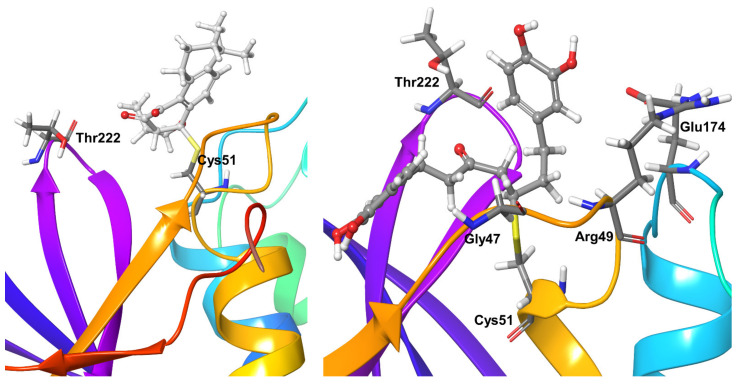
Stable interactions observed during MD refinement simulations of top poses of Compound **3** (**left**) and **4** (**right**) with OTUB-2. The ligands are shown in ball and stick representation, stable and moderately stable protein-ligand interacting residues are represented as thick tubes, short-living interactions (present for less than 30% of simulation time) are represented as thin tubes.

The covalent inhibitor Compound **3** also formed short-range hydrophobic interactions with targeted residue Cys51 plus very short water-mediated interactions. Except for Thr222, for which water-mediated hydrogen bonds could be observed for 30% of the simulation time, no other significant interactions were found (Figure 11).

Compound **4** only formed hydrophobic interactions with Cys51 from OTUB2 during the entire simulations. The protein residue Arg49 showed direct and water-mediated hydrogen bond interactions for nearly 60% of the simulation time with compound **4**. Gly47, Glu174, and Thr222 showed a mixture of direct and water-mediated hydrogen bond interactions with the compound for 30–40% of the simulation time (Figure 11).

**Figure 12:**
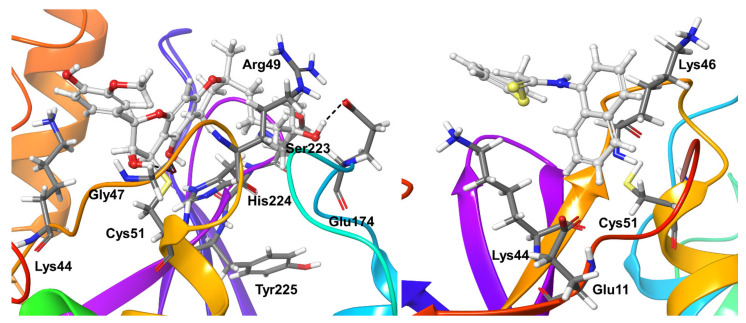
Stable ligand-protein interactions during MD refinement simulations of top poses of Compound **5** (**left**) and **6** (**right**) with OTUB-2. The compounds are shown in ball and stick representation, stable and moderately stable protein-ligand interactions are represented as thick tubes, and short-living interactions (present for less than 30% of simulation time) are shown as thin tubes.

Compound **5** appeared as a stronger and less flexible OTUB2 binder. It formed long-living and more interactions with the OTUB2 protein. Apart from the previously discussed hydrophobic interaction with the covalently-connected residue Cys51, Tyr225 maintained the hydrogen bond interactions (both direct and water-mediated) with the ligand during the entire simulations. In addition, OTUB2 residues Lys44, Gly47, Arg49, Glu174, Ser223, and His224 showed a mixture of direct and water-mediated hydrogen bond interactions for nearly 40–80% of the simulation time. In addition, His224 exhibited hydrophobic interactions for a significant time during simulations (Figure 12). 

Compound **6** appeared as a weak binder and only showed interactions with OTUB2 residue Glu11 for around 35% of the simulation time; no other significant protein-ligand interactions were found. Two lysine residues Lys44 and Lys46, showed shorter living hydrophobic interactions for 15–20% of the simulation time (Figure 12).

The RMSD plots of all the six compounds with all three proteins were given in the Supplementary Material (Figures S12–S20).

### 2.6. Molecular Dynamics Refinement of Covalent SARS-CoV-2 PLpro Inhibitors

Recently, the SARS-CoV-2 PLpro X-ray structure with two covalent inhibitors (PDB entries 6 WUU and 6WX4) was reported [28]. These peptidomimetic ligands were not of therapeutic applicability but revealed exciting details about protein-ligand interactions.

We refined these structures using the chosen MD protocol. The complex of PLpro with the peptide inhibitors **VIR250** at 2.79 Å (6 WUU) and **VIR251** at 1.67 Å resolution were covalent ligand-protein complexes with short cysteine-ligand distances of 1.88 A and 1.79 Å, respectively [28]. Cys111 underwent a Michael addition to the Cβ of the VMW warhead and forms a covalent thioether bond.

The two inhibitors were spatially extended peptidomimetics with unnatural amino acids Ac-Abu(Bth)-Dap-Gly-Gly-VME (**VIR250**) and Ac-hTyr-Dap-Gly-Gly-VME (**VIR251**) type. The **VIR250** and **VIR251** inhibitors occupy the S4-S1 pocket of CoV-2 PLpro protein in proximity to the active site, and both ligands adopt similar conformations (see Figure S21).

The ligand RMSD from the MD simulation results of 6 WUU and 6WX4 were 1.14 Å and 1.41 Å, respectively. MD refinement of the 6WUU **VIR250** structure showed that the crystal structure ligand-protein hydrogen bonds with Gly163 and Gly271 and those with two tyrosine residues (Tyr 268 and Tyr 273) were retained during the MD refinement simulations (Figure S22). Apart from these direct hydrogen bonds, water-mediated interactions were also observed with residues Asp164 and Arg166. As in the OTU simulations, the active site residue Cys111 undergoes persistent hydrophobic interactions with the peptidic inhibitors. For **VIR251** in the crystal structure 6WX4, MD simulations revealed stable hydrogen bonding interactions of the ligand with Gly163, Tyr264, and Gly271. Hydrophobic interactions with Cys111 and Pro248 and water-mediated interactions with the residue Asp164 contributed to the ligand stability. 

## 3. Materials and Methods

### 3.1. Protein Structures and Preparation

For OTUB1, OTUB2, and SARS-CoV-2, the PDB entries 2ZFY, 1TFF, and 6 WUU were used. Covalently-bound OTUB2 structures [16] were PDB entries 5QIP, 5QIQ, 5QIR, 5QIS, 5QIT, 5QIU, 5QIV, 5QIW, 5QIX, 5QIY, 5QIZ. The protein RMSD between the apo and the ligand-bound OTUB2 structures was 0.6 Å and showed that ligand binding did not induce large conformation re-arrangements.

The protein structures were prepared by removing steric clashes, co-crystallized additives and refined by optimizing side-chain orientations and water molecule positions using the protein preparation wizard [29] and Epik [30,31]. Bond orders were automatically assigned, hydrogen atoms added, and side-chain protonation states were assigned using PROPKA at pH 7.0. The protein was minimized using the OPLS2005 forcefield. The ligands were prepared using LigPrep [32] and the OPLS2005 forcefield, ionized to identify possible states at target pH using 7.0 ± 2.0. Unlike commonly used forcefield types, no additional topology files needed to be provided. Desmond automatically assigns distances, angles, dihedral, and other parameters from the structure file (.mae).

### 3.2. Covalent and Non-Covalent Ligand Docking

Covalent ligand docking is a multi-step process [17] starting from a standard non-covalent docking with the reactive amino acid residues replaced by alanine to incorporate the ligands. The receptor active site cysteine was selected as a reactive residue, and the grid box was positioned at the centroid of the active site residues. The pre-reaction ligands were docked with Glide [33,34,35] to generate guess poses suitable for covalent bond formation. ConfGen [36] was used to sample ligand conformations before covalent docking upon mutating the reactive residue to alanine. This allowed to position the warhead close to the targeted cysteine and avoid unfavorable clashes. The mutation was then reversed, and rotamer states of the nucleophilic cysteine residue were sampled to form a covalent bond with different top-scoring non-covalent ligand poses based on geometric criteria. The docked position was selected as a pose prediction (in ‘Thorough Mode’) with the cutoff of 2.5 kcal/mol and positional constraints for pose selections. After the initial docking of the ligand with mutated alanine, the reactive cysteine side chain was regenerated, and a ConfGen rotamer sampling was performed to identify the best side-chain conformation for each docked ligand pose. The covalent ligand-cysteine linkage specified by the reaction type was formed, and further minimization and clustering of poses were carried out. The representative poses from the cluster were minimized. Based on the reaction chemistry of the compounds, we have used Michael addition, or Nucleophilic addition to a double bond was used. The Covdock score, which was defined as the average between the Glide score of the binding mode of the pre-reactive ligand and the ligand score in the final covalent complex, was employed.

For non-covalent docking, Glide XP [35] docking was used with default parameters, and allowed intramolecular hydrogen bonds were rewarded. Interaction scores concerning every residue within 6 Å of the grid center were calculated. The grid was generated before docking with the centroid at the active site residues and outer box dimension of 30 Å × 30 Å × 30 Å.

### 3.3. Molecular Dynamics Simulations

Following molecular docking, 3 independent 100 ns Molecular Dynamics simulations were performed for each covalent and non-covalent ligand-protein complex using the GPU-accelerated Desmond software version 2020-4 [37]. The ligand-protein complexes were solvated using the TIP3P water model [38,39]. Periodic boundary conditions were set, and the box’s volume was minimized after including the protein and solvent. A respective number of ions was added to neutralize the system before simulation at a physiological salt concentration of 150 mM sodium chloride. 

All MD simulations were performed using Desmond with a time step of 2 fs in triplicates of 100 ns to refine the ligand binding positions. Trajectory recording was performed in intervals of 10 ps, in an NPT ensemble at 300 K temperature using the Nose–Hoover chain thermostat, and 1 bar atmospheric pressure was maintained using a Martyna–Tobias–Klein barostat. An equilibration time of 10 ns was used to ensure that the system was equilibrated before the production runs. A cutoff radius of 9.0 Å controlled short-range electrostatic interactions. Long-range Coulomb interactions were treated using the particle mesh Ewald (PME) method. No positional restraints were used.

Desmond used an intrinsic automatic topology builder for the OPLS2005 force field to generate parameters based on the input file (atom and bond types). There were no distance constraints used in this study. The covalent bond parameters were taken from the OPLS2005 force field. Desmond automatically assigns distances, angles, and dihedral parameters from the structure files (.mae) and atomic charges. MD interaction analysis was carried out as discussed in our previous works [15,40,41].

## 4. Conclusions

Deubiquitinating enzymes have not only recently received attention as drug targets in cancer, autoimmune diseases, chronic inflammation, and neurodegeneration, but the functionally related papain-like protease PLpro may also be a viable target against SARS-CoV-2. First selective peptidomimetic covalent inhibitors of the OTUB2 DUB show that this class of drug compounds could irreversibly inhibit the cysteine of the catalytic triad. The molecular dynamics refinement of covalently bound inhibitors gives additional insight into the inhibitor-protein interactions and their persistence over time. The simulations well reproduce structural features of the covalent ligand-protein binding.

The sequence identity of 83% and 90% similarity between the PLpro from SARS-CoV and SARS-CoV-2 indicates a similar overall structure and accessibility of the active site for non-covalent and covalent inhibitors. The binding of a subset of known PLpro SARS-CoV inhibitors demonstrates that they may also be accommodated in the SARS-CoV-2 PLpro. The binding of the SARS-CoV inhibitors to PLpro is significantly preferred over OTUB1 and OTUB2. Based on the interactions of the protein active site residues with the inhibitor ligand, we can identify several critical protein-ligand binding interactions regarding SARS-CoV and SARS-CoV-2 PLpro but also human DUBs OTUB1 and OTUB2. Despite an overall similarity on the sequence and structural level, these differences in the vicinity of the active sites regulate the accessibility and binding of inhibitor molecules.

In vitro studies have recently shown that a re-purposing strategy using 3727 unique known drugs towards SARS-CoV-2 PLpro is unlikely to yield suitable drug candidates and highlights the importance of a counter screen in assessing the validity of hits coming from one screen of known drugs before any conclusions regarding their therapeutic potential can be drawn [14]. Targeting the SARS-CoV-2 PLpro by re-purposing of known therapeutics does not appear promising.

The potential of the development of novel irreversible covalent inhibitors to the catalytic cysteine or strong non-covalent blocking its accessibility has to be exploited. It requires a joint effort of medicinal chemists and structural biologists to design suitable compounds with reactive ‘warheads’ and pharmacologists to assay their inhibitory effects; such work is currently ongoing in our lab.

## Figures and Tables

**Table 1:** Binding of covalent and non-covalent SARS-CoV PLpro inhibitors to SARS-CoV-2 PLpro, OTUB1, and OTUB2. Analysis of relevant hydrogen bonding and hydrophobic protein-ligand interactions.

Compound	Target Protein	Hydrogen Bond Interactions	Hydrophobic Interactions *
**1**	SARS-CoV-2 PLpro	-	-
	OTUB1	Pro1263	**His1265**
	OTUB2	Thr222, **Asn226**	**His224**
**2**	SARS-CoV2 PLpro	Ala288	-
	OTUB1	Glu1060, Pro1263	-
	OTUB2	Lys221, Thr222, **Asn226**	**His224**
**3**	SARS-CoV2 PLpro	**Cys111**	
	OTUB1	-	-
	OTUB2	-	-
**4**	SARS-CoV2 PLpro	**Cys111**, Gly163, Tyr268	Tyr264, **His272**
	OTUB1	Pro1087, Asp1216	Tyr1261
	OTUB2	Thr45, Gly47	**His224**
**5**	SARS-CoV2 PLpro	**Cys111**, Tyr112, Tyr264	Tyr264, **His272**
	OTUB1	Gly1264	-
	OTUB2	Arg49, Ser223	-
**6**	SARS-CoV2 PLpro	-	**His272**, Trp106
	OTUB1	Glu1060	-
	OTUB2	Thr45	-

* Mostly π-π interactions. Active site residues are given in bold.

## Data Availability

The data presented in this study are available in the article and Supplementary Materials.

## Supplementary Materials

The following data are available online at https://www.mdpi.com/article/10.3390/biom11060802/s1, Figure S1: Dominating molecular interactions observed during the molecular dynamics simulation of the complex Otub-2 with compound PCM-0102153 (benzyl acetylcarbamate) (Pdb id 5QIP). The covalent ligand is represented as ball and sticks and the interacting residues as sticks. Figure S2. Dominating molecular interaction observed during the molecular dynamics simulation of the complex Otub-2 with compound PCM-0103050 (*N*-[(4-bromothiophen-2-yl)-methyl]acetamide-pdb id 5QIQ. The covalent ligand is represented as ball and sticks and the interacting residues as sticks. Figure S3. Dominating molecular interaction observed during the molecular dynamics simulation of the complex Otub-2 with compound PCM-0102305 (*N*-[(4-fluoro-3-methylphenyl)-methyl]acetamide-pdb id 5QIR. The covalent ligand is represented as ball and sticks and the interacting residues as sticks. Figure S4. Dominating molecular interaction observed during the molecular dynamics simulation of the complex Otub-2 with compound PCM-0102500 (*N*-(5-methyl-1,2-oxazol-3-yl)acetamide-pdb id 5QIS. The covalent ligand is represented as ball and sticks and the interacting residues as sticks. Figure S5. Dominating molecular interaction observed during the molecular dynamics simulation of the complex Otub-2 with compound PCM-0102821 *N*-[(E)-(3-methylphenyl) methylidene] acetamide-pdb id 5QIT. The covalent ligand is represented as ball and sticks and the interacting residues as sticks. Figure S6. Dominating molecular interaction observed during the molecular dynamics simulation of the complex Otub-2 with compound PCM-0103011 *N*-{3-[3-(trifluoromethyl)phenyl]prop-2-yn-1-yl}acetamide-pdb id 5QIU. The covalent ligand is represented as ball and sticks and the interacting residues as sticks. Figure S7. Dominating molecular interaction observed during the molecular dynamics simulation of the complex Otub-2 with compound PCM-0102998 *N*′-acetyl-2-[(3R)-1,1-dioxo-1lambda~6~-thiolan-3-yl] acetohydrazide-pdb id 5QIV. The covalent ligand is represented as ball and sticks and the interacting residues as sticks. Figure S8. Dominating molecular interaction observed during the molecular dynamics simulation of the complex Otub-2 with compound PCM-0102660 *N*-[(E)-(4-methylphenyl)methylidene] acetamide-pdb id 5QIW. The covalent ligand is represented as ball and sticks and the interacting residues as sticks. Figure S9. Dominating molecular interaction observed during the molecular dynamics simulation of the complex Otub-2 with compound PCM-0103007 *N*-(3-phenylprop-2-yn-1-yl)acetamid-pdb id 5QIX. The covalent ligand is represented as ball and sticks and the interacting residues as sticks. Figure S10. Dominating molecular interaction observed during the molecular dynamics simulation of the complex Otub-2 with compound PCM-0102954 *N*′-acetyl-2-chlorobenzohydrazide-pdb id 5QIY. The covalent ligand is represented as ball and sticks and the interacting residues as sticks. Figure S11. Dominating molecular interactions observed during the molecular dynamics simulation of the complex Otub-2 with compound PCM-0103080 (N-[(5-chlorothiophen-2-yl) methyl] acetamide) - pdb id 5QIZ. The covalent ligand is represented as ball and sticks and the interacting residues as sticks. Table S1. Covalent selective inhibitors of the OTUB2 DUB. Comparison of co-crystallized ligand with MD refined structures. Figure S12. Molecular Dynamics studies of SARS-Cov-2 PlPro to show the RMSD of protein and Ligand over time for Compound **1** (left) and Compound **2** (right). Figure S13. Molecular Dynamics studies of SARS-Cov-2 PlPro to show the RMSD of protein and Ligand over time for Compound **3** (left) and Compound **4** (right). Figure S14. Molecular Dynamics studies of SARS-Cov-2 PLPro to show the RMSD of protein and Ligand over time for Compound **5** (left) and Compound **6** (right). Figure S15. Molecular Dynamics studies of OTUB-1 to show the RMSD of protein and Ligand over time for Compound **1** (left) and Compound **2** (right). Figure S16. Molecular Dynamics studies of OTUB-1 to show the RMSD of protein and Ligand over time for Compound **3** (left) and Compound **4** (right). Figure S17. Molecular Dynamics studies of OTUB-1 to show the RMSD of protein and Ligand over time for Compound **5** (left) and Compound **6** (right). Figure S18. Molecular Dynamics studies ofOTUB-2 to show the RMSD of protein and Ligand over time for Compound **1** (left) and Compound **2** (right). Figure S19. Molecular Dynamics studies of OTUB-2 to show the RMSD of protein and Ligand over time for Compound **3** (left) and Compound **4** (right). Figure S20. Molecular Dynamics studies of OTUB-2 to show the RMSD of protein and Ligand over time for Compound **5** (left) and Compound **6** (right). Figure S21. Cov-2-PLpro with its inhibitors in the X-ray structures (ligand carbon in grey) and refined with MD simulations (ligand carbon in green). (A) 6WUU with peptide inhibitor VIR250 and B) 6WX4 with peptide inhibitor VIR251.Figure S22. Dominating non-covalent protein-ligand interactions of the peptide inhibitors (A) VIR250, (B) VIR251 with the PLpro SARS-CoV-2 during MD refinement simulations. Persistence of interactions is given in percent of simulation time.

## References

[1] Xia S., Liu M., Wang C., Xu W., Lan Q., Feng S., Qi F., Bao L., Du L., Liu S. (2020). Inhibition of SARS-CoV-2 (Previously 2019-NCoV) Infection by a Highly Potent Pan-Coronavirus Fusion Inhibitor Targeting Its Spike Protein That Harbors a High Capacity to Mediate Membrane Fusion. Cell Res..

[2] Zhang L., Lin D., Sun X., Curth U., Drosten C., Sauerhering L., Becker S., Rox K., Hilgenfeld R. (2020). Crystal Structure of SARS-CoV-2 Main Protease Provides a Basis for Design of Improved α-Ketoamide Inhibitors. Science.

[3] Kneller D.W., Phillips G., O’Neill H.M., Jedrzejczak R., Stols L., Langan P., Joachimiak A., Coates L., Kovalevsky A. (2020). Structural Plasticity of SARS-CoV-2 3CL M pro Active Site Cavity Revealed by Room Temperature X-Ray Crystallography. Nat. Commun..

[4] Kandeel M., Al-Nazawi M. (2020). Virtual Screening and Re-purposing of FDA Approved Drugs against COVID-19 Main Protease. Life Sci..

[5] Ratia K., Pegan S., Takayama J., Sleeman K., Coughlin M., Baliji S., Chaudhuri R., Fu W., Prabhakar B.S., Johnson M.E. (2008). A Noncovalent Class of Papain-like Protease/Deubiquitinase Inhibitors Blocks SARS Virus Replication. Proc. Natl. Acad. Sci. USA.

[6] Van Kasteren P.B., Bailey-Elkin B.A., James T.W., Ninaber D.K., Beugeling C., Khajehpour M., Snijder E.J., Mark B.L., Kikkert M. (2013). Deubiquitinase Function of Arterivirus Papain-like Protease 2 Suppresses the Innate Immune Response in Infected Host Cells. Proc. Natl. Acad. Sci. USA.

[7] Ratia K., Kilianski A., Baez-Santos Y.M., Baker S.C., Mesecar A. (2014). Structural Basis for the Ubiquitin-Linkage Specificity and DeISGylating Activity of SARS-CoV Papain-like Protease. PLoS Pathog..

[8] Báez-Santos Y.M., St. John S.E., Mesecar A.D. (2015). The SARS-Coronavirus Papain-like Protease: Structure, Function and Inhibition by Designed Antiviral Compounds. Antiviral Res..

[9] Shagufta, Ahmad I. (2021). The Race to Treat COVID-19: Potential Therapeutic Agents for the Prevention and Treatment of SARS-CoV-2. Eur. J. Med. Chem..

[10] Smith M., Smith J.C. (2020). Repurposing Therapeutics for COVID-19: Supercomputer-Based Docking to the SARS-CoV-2 Viral Spike Protein and Viral Spike Protein-Human ACE2 Interface. J. Chem. Inf. Model..

[11] Wu C., Liu Y., Yang Y., Zhang P., Zhong W., Wang Y., Wang Q., Xu Y., Li M., Li X. (2020). Analysis of Therapeutic Targets for SARS-CoV-2 and Discovery of Potential Drugs by Computational Methods. Acta Pharm. Sin. B.

[12] Arya R., Das A., Prashar V., Kumar M. (2020). Potential Inhibitors against Papain-like Protease of Novel Coronavirus (SARS-CoV-2) from FDA Approved Drugs. ChemRxiv.

[13] Shin D., Mukherjee R., Grewe D., Bojkova D., Baek K., Bhattacharya A., Schulz L., Widera M., Mehdipour A.R., Tascher G. (2020). Papain-like Protease Regulates SARS-CoV-2 Viral Spread and Innate Immunity. Nature.

[14] Klemm T., Ebert G., Calleja D.J., Allison C.C., Richardson L.W., Bernardini J.P., Lu B.G., Kuchel N.W., Grohmann C., Shibata Y. (2020). Mechanism and Inhibition of the Papain-like Protease, PLpro, of SARS-CoV-2. EMBO J..

[15] Sivakumar D., Kumar V., Naumann M., Stein M. (2020). Activation and Selectivity of OTUB-1 and OTUB-2 Deubiquitinylases. J. Biol. Chem..

[16] Resnick E., Bradley A., Gan J., Douangamath A., Krojer T., Sethi R., Geurink P.P., Aimon A., Amitai G., Bellini D. (2019). Rapid Covalent-Probe Discovery by Electrophile-Fragment Screening. J. Am. Chem. Soc..

[17] Zhu K., Borrelli K.W., Greenwood J.R., Day T., Abel R., Farid R.S., Harder E. (2014). Docking Covalent Inhibitors: A Parameter Free Approach to Pose Prediction and Scoring. J. Chem. Inf. Model..

[18] Klein P., Barthels F., Johe P., Wagner A., Tenzer S., Distler U., Le T.A., Schmid P., Engel V., Engels B. (2020). Naphthoquinones as Covalent Reversible Inhibitors of Cysteine Proteases—Studies on Inhibition Mechanism and Kinetics. Molecules.

[19] Palmer J.T., Rasnick D., Klaus J.L., Brömme D. (1995). Vinyl Sulfones as Mechanism-Based Cysteine Protease Inhibitors. J. Med. Chem..

[20] Johansson M.H. (2012). Reversible Michael Additions: Covalent Inhibitors and Prodrugs. Mini Rev. Med. Chem..

[21] Ettari R., Micale N., Schirmeister T., Gelhaus C., Leippe M., Nizi E., Di Francesco M.E., Grasso S., Zappalà M. (2009). Novel Peptidomimetics Containing a Vinyl Ester Moiety as Highly Potent and Selective Falcipain-2 Inhibitors. J. Med. Chem..

[22] Chen X., Chou C.-Y., Chang G.-G. (2009). Thiopurine Analogue Inhibitors of Severe Acute Respiratory Syndrome-Coronavirus Papain-like Protease, a Deubiquitinating and DeISGylating Enzyme. Antivir. Chem. Chemother..

[23] Chou C.-Y., Chien C.-H., Han Y.-S., Prebanda M.T., Hsieh H.-P., Turk B., Chang G.-G., Chen X. (2008). Thiopurine Analogues Inhibit Papain-like Protease of Severe Acute Respiratory Syndrome Coronavirus. Biochem. Pharmacol..

[24] Park J.-Y., Kim J.H., Kim Y.M., Jeong H.J., Kim D.W., Park K.H., Kwon H.-J., Park S.-J., Lee W.S., Ryu Y.B. (2012). Tanshinones as Selective and Slow-Binding Inhibitors for SARS-CoV Cysteine Proteases. Bioorg. Med. Chem..

[25] Park J.-Y., Jeong H.J., Kim J.H., Kim Y.M., Park S.-J., Kim D., Park K.H., Lee W.S., Ryu Y.B. (2012). Diarylheptanoids from Alnus Japonica Inhibit Papain-like Protease of Severe Acute Respiratory Syndrome Coronavirus. Biol. Pharm. Bull..

[26] Jk C., Mj C.-L., Kh L., Dw K., Hw R., Hj Y., Kh P. (2013). Geranylated Flavonoids Displaying SARS-CoV Papain-like Protease Inhibition from the Fruits of Paulownia Tomentosa. Bioorg. Med. Chem..

[27] Frieman M., Basu D., Matthews K., Taylor J., Jones G., Pickles R., Baric R., Engel D.A. (2011). Yeast Based Small Molecule Screen for Inhibitors of SARS-CoV. PLoS ONE.

[28] Rut W., Lv Z., Zmudzinski M., Patchett S., Nayak D., Snipas S.J., Oualid F.E., Huang T.T., Bekes M., Drag M. (2020). Activity Profiling and Crystal Structures of Inhibitor-Bound SARS-CoV-2 Papain-like Protease: A Framework for Anti–COVID-19 Drug Design. Sci. Adv..

[29] Sastry G.M., Adzhigirey M., Day T., Annabhimoju R., Sherman W. (2013). Protein and Ligand Preparation: Parameters, Protocols, and Influence on Virtual Screening Enrichments. J. Comput. Aided Mol. Des..

[30] Greenwood J.R., Calkins D., Sullivan A.P., Shelley J.C. (2010). Towards the Comprehensive, Rapid, and Accurate Prediction of the Favorable Tautomeric States of Drug-like Molecules in Aqueous Solution. J. Comput. Aided Mol. Des..

[31] Shelley J.C., Cholleti A., Frye L.L., Greenwood J.R., Timlin M.R., Uchimaya M. (2007). Epik: A Software Program for PK(a) Prediction and Protonation State Generation for Drug-like Molecules. J. Comput. Aided Mol. Des..

[32] (2021). Schrödinger Release 2021-1: LigPrep.

[33] Friesner R.A., Banks J.L., Murphy R.B., Halgren T.A., Klicic J.J., Mainz D.T., Repasky M.P., Knoll E.H., Shelley M., Perry J.K. (2004). Glide: A New Approach for Rapid, Accurate Docking and Scoring. 1. Method and Assessment of Docking Accuracy. J. Med. Chem..

[34] Halgren T.A., Murphy R.B., Friesner R.A., Beard H.S., Frye L.L., Pollard W.T., Banks J.L. (2004). Glide: A New Approach for Rapid, Accurate Docking and Scoring. 2. Enrichment Factors in Database Screening. J. Med. Chem..

[35] Friesner R.A., Murphy R.B., Repasky M.P., Frye L.L., Greenwood J.R., Halgren T.A., Sanschagrin P.C., Mainz D.T. (2006). Extra Precision Glide:  Docking and Scoring Incorporating a Model of Hydrophobic Enclosure for Protein−Ligand Complexes. J. Med. Chem..

[36] Watts K.S., Dalal P., Murphy R.B., Sherman W., Friesner R.A., Shelley J.C. (2010). ConfGen: A Conformational Search Method for Efficient Generation of Bioactive Conformers. J. Chem. Inf. Model..

[37] Bowers K.J., Chow E., Xu H., Dror R.O., Eastwood M.P., Gregersen B.A., Klepeis J.L., Kolossvary I., Moraes M.A., Sacerdoti F.D. (2006). Scalable Algorithms for Molecular Dynamics Simulations on Commodity Clusters. Proceedings of the 2006 ACM/IEEE Conference on Supercomputing.

[38] Ash J., Fourches D. (2017). Characterizing the Chemical Space of ERK2 Kinase Inhibitors Using Descriptors Computed from Molecular Dynamics Trajectories. J. Chem. Inf. Model..

[39] Kaczor A.A., Targowska-Duda K.M., Patel J.Z., Laitinen T., Parkkari T., Adams Y., Nevalainen T.J., Poso A. (2015). Comparative Molecular Field Analysis and Molecular Dynamics Studies of α/β Hydrolase Domain Containing 6 (ABHD6) Inhibitors. J. Mol. Model..

[40] Sivakumar D., Gorai B., Sivaraman T. (2013). Screening Efficient BH3-Mimetics to HBcl-B by Means of Peptidodynmimetic Method. Mol. BioSyst..

[41] Sivakumar D., Mudedla S., Jang S., Kim H., Park H., Choi Y., Oh J., Wu S. (2021). Computational Study on Selective PDE9 Inhibitors on PDE9-Mg/Mg, PDE9-Zn/Mg, and PDE9-Zn/Zn Systems. Biomolecules.

