# Supplementary material for: Binding of SARS-CoV Covalent Non-Covalent Inhibitors to the SARS-CoV-2 Papain-Like Protease and Ovarian Tumor Domain Deubiquitinases
Source: Biomolecules. 2021 May 28;11(6):802. doi: 10.3390/biom11060802 (PMC8227062; doi:10.3390/biom11060802)
Supplement: Supplementary file 1 [file biomolecules-11-00802-s001.zip › biomolecules-1167124-supplementary.pdf]

Supplementary information

Binding of SARS-CoV Covalent Inhibitors to the SARS-CoV2 Papain-like Protease and Ovarian Tumor Domain Deubiquitinases

Dakshinamurthy Sivakumar † and Matthias Stein *

Molecular Simulations and Design Group, Max Planck Institute for Dynamics of Complex Technical Systems, University, Sandtorstrasse 1, 39106 Magdeburg, Germany; sivakumar@mpi-magdeburg.mpg.de

* Correspondence: matthias.stein@mpi-magdeburg.mpg.de

† Current address: R&D Center, Pharmcadd, 221, 17 APEC-ro, Haeundae-gu, Busan 48060, Korea

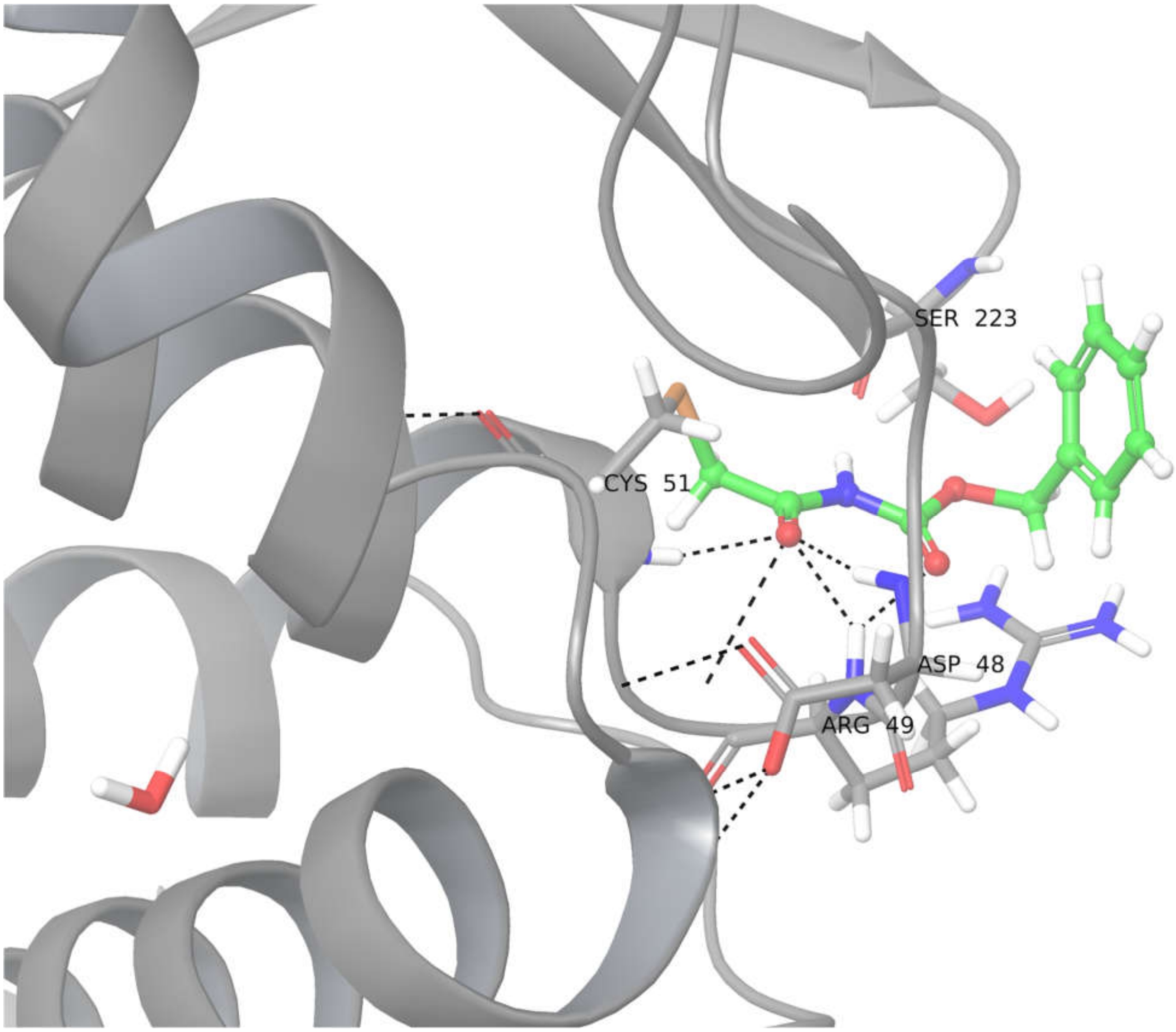

Figure S1. Dominating molecular interactions observed during the molecular dynamics simulation of the complex Otub-2 with compound PCM-0102153 (benzyl acetylcarbamate) (Pdb id 5QIP). The covalent ligand is represented as ball and sticks and the interacting residues as sticks.

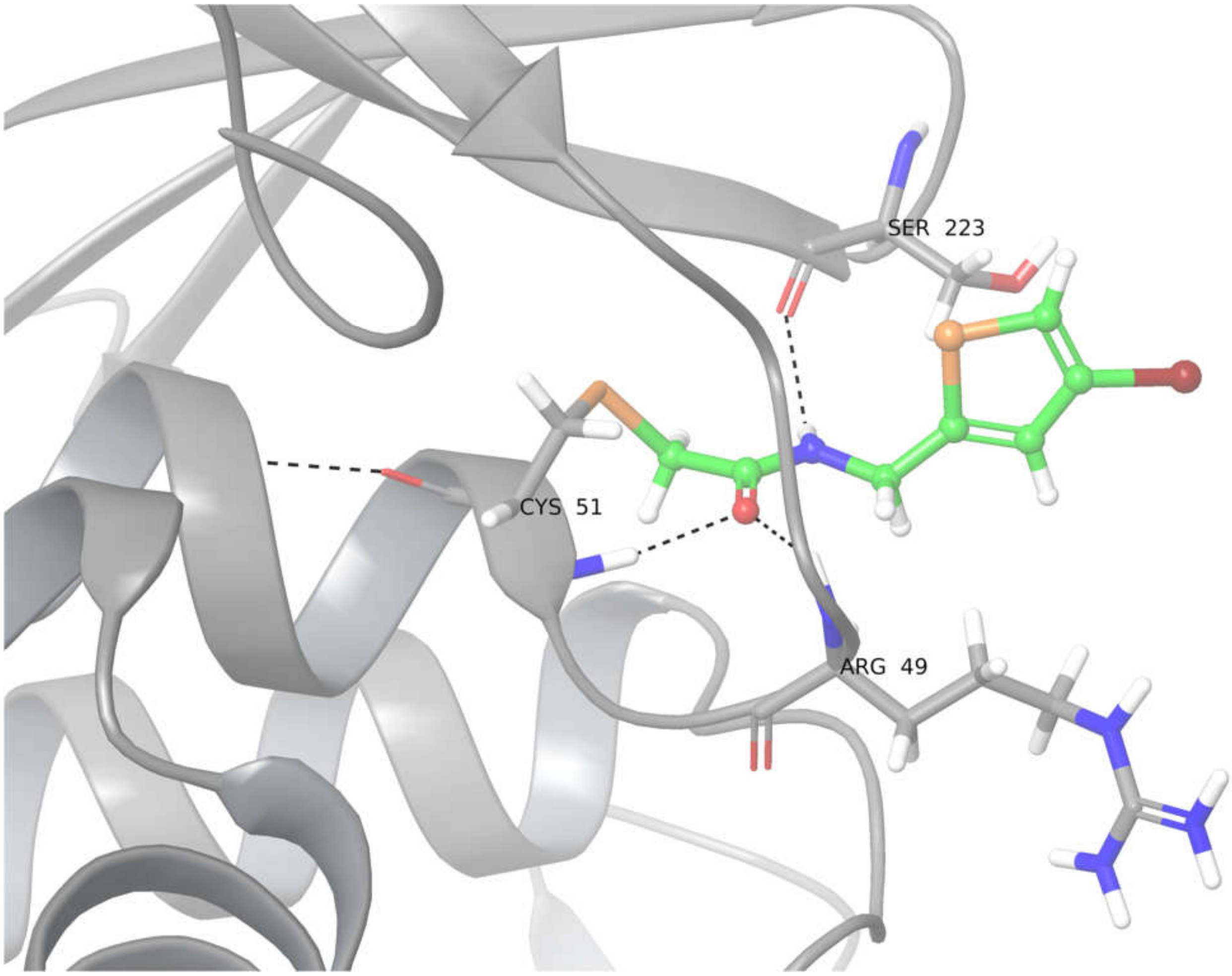

Figure S2. Dominating molecular interaction observed during the molecular dynamics simulation of the complex Otub-2 with compound PCM-0103050 (N-[(4-bromothiophen-2-yl)-methyl] acetamide) - pdb id 5QIQ. The covalent ligand is represented as ball and sticks and the interacting residues as sticks.

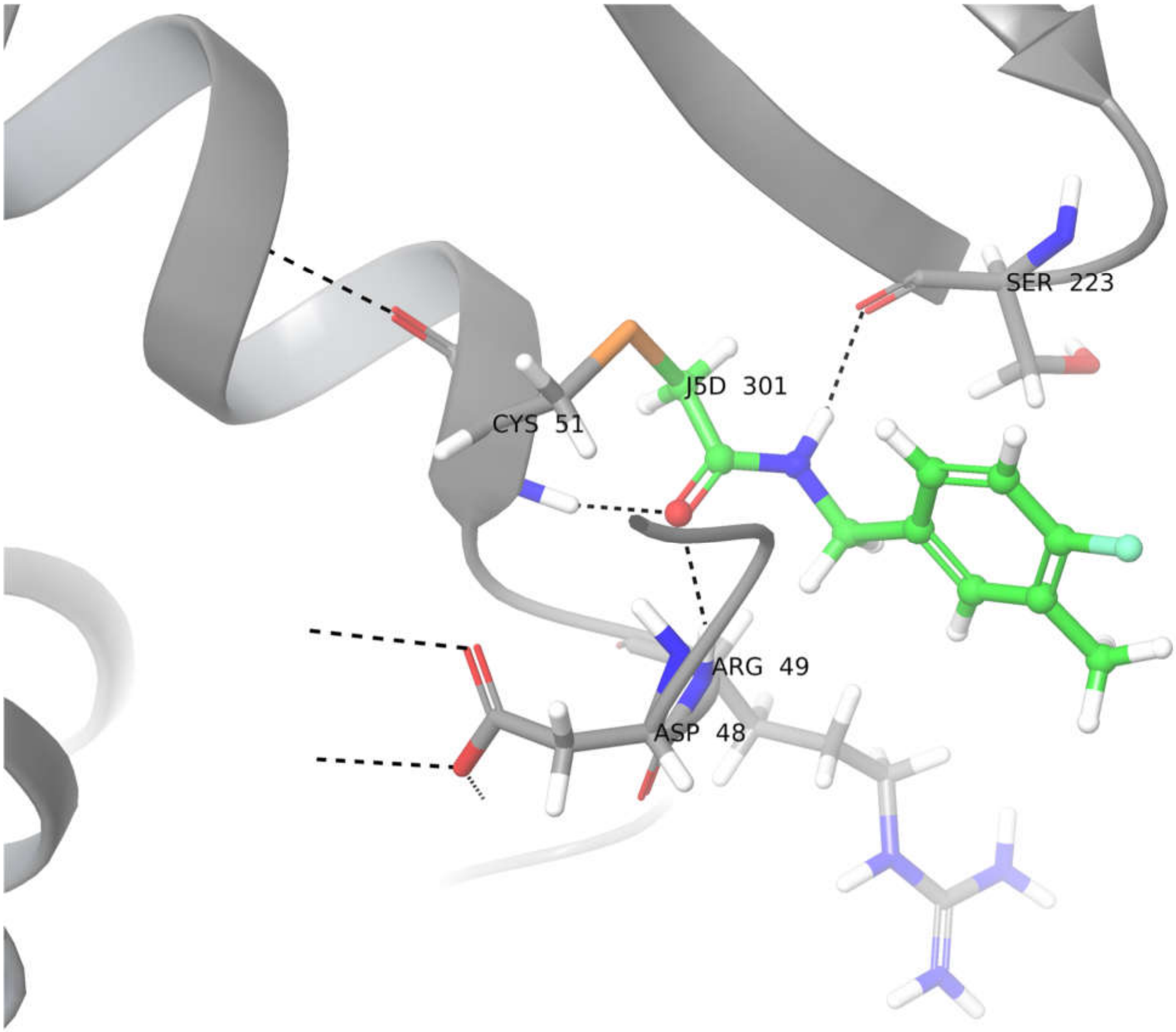

Figure S3. Dominating molecular interaction observed during the molecular dynamics simulation of the complex Otub-2 with compound PCM-0102305 (N-[(4-fluoro-3-methylphenyl)-methyl] acetamide) - pdb id 5QIR. The covalent ligand is represented as ball and sticks and the interacting residues as sticks.

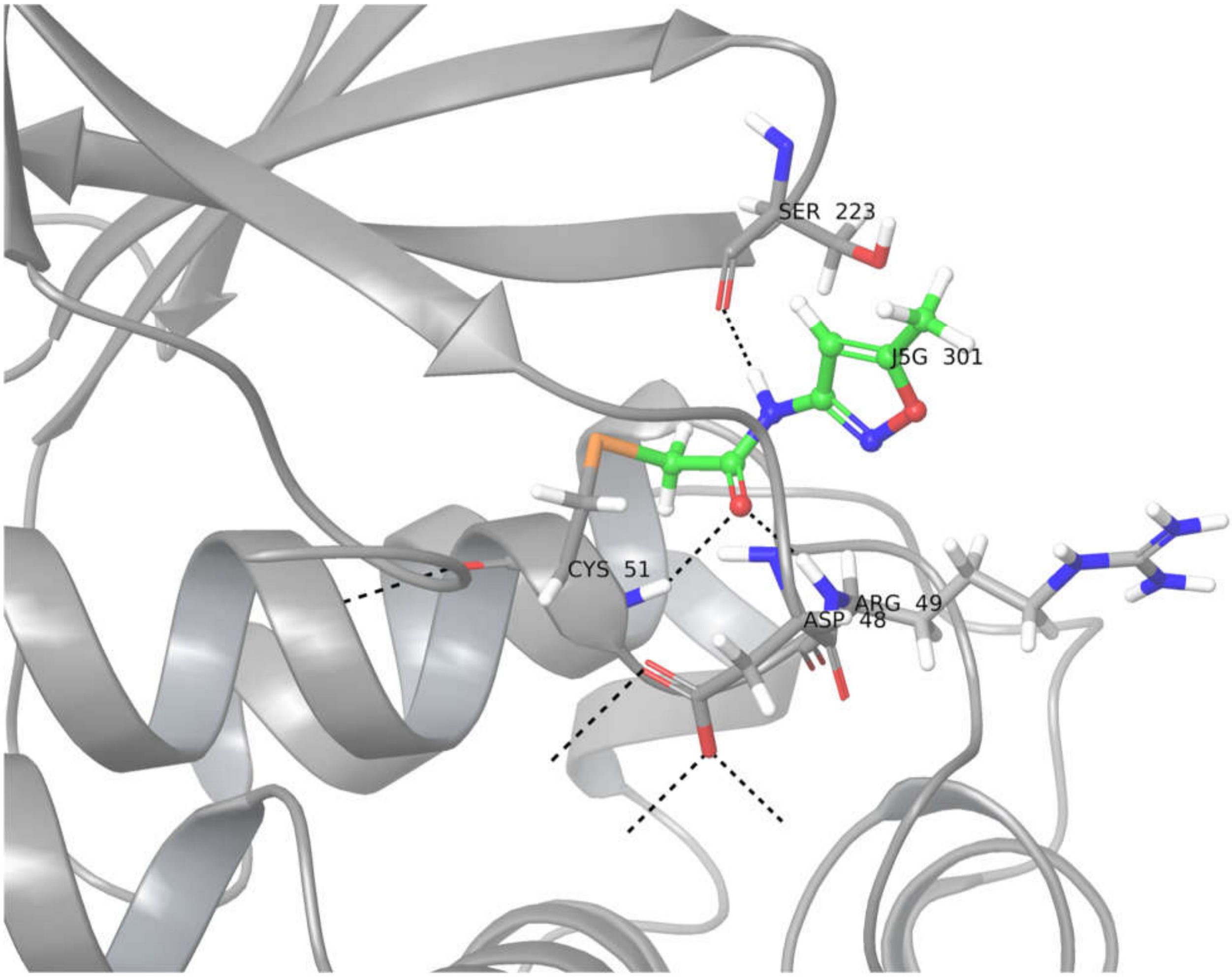

Figure S4. Dominating molecular interaction observed during the molecular dynamics simulation of the complex Otub-2 with compound PCM-0102500 (N-(5-methyl-1,2-oxazol-3-yl)acetamide) - pdb id 5QIS. The covalent ligand is represented as ball and sticks and the interacting residues as sticks.

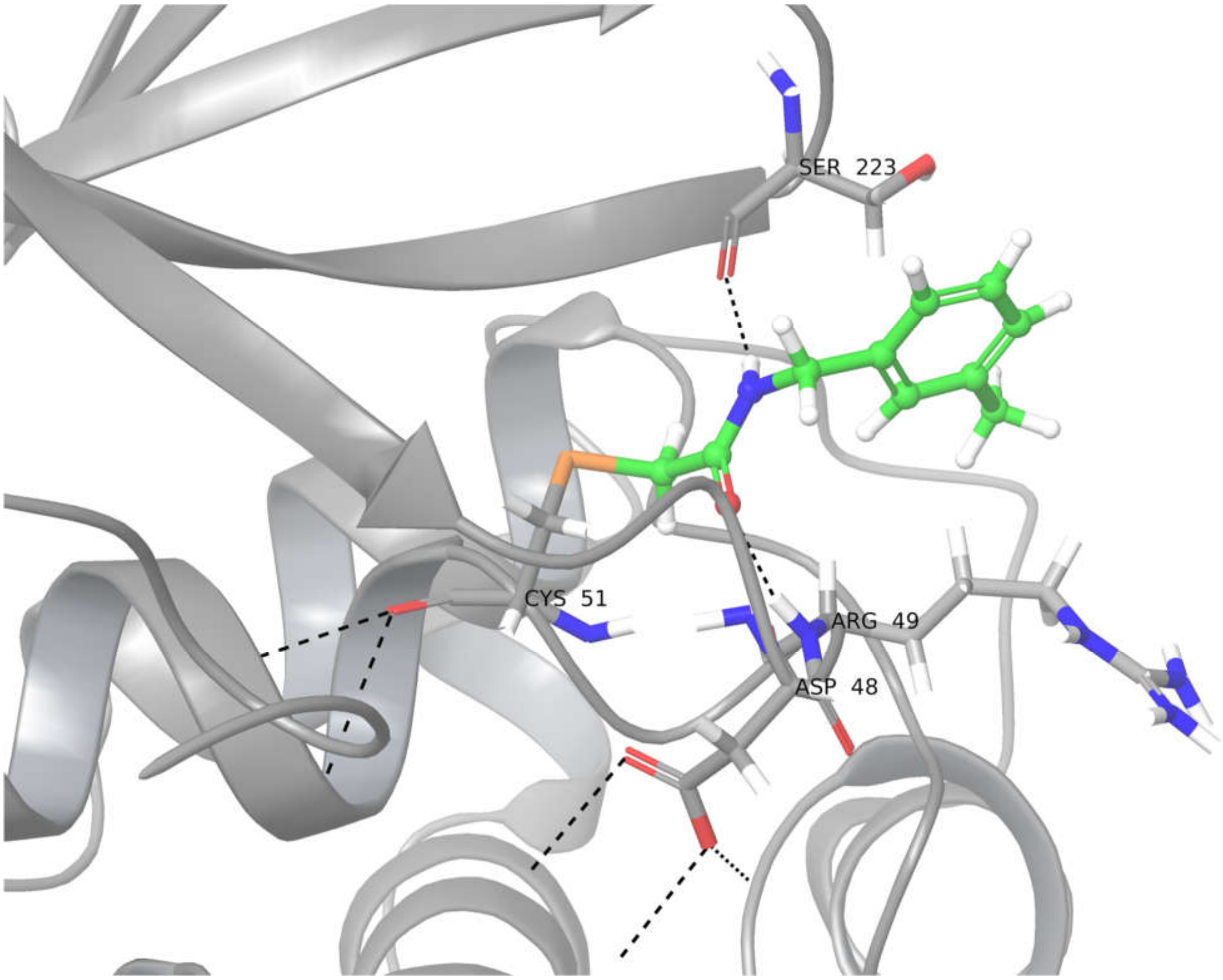

Figure S5. Dominating molecular interaction observed during the molecular dynamics simulation of the complex Otub-2 with compound PCM-0102821 (N-[(E)-(3-methylphenyl) methylidene] acetamide) - pdb id 5QIT. The covalent ligand is represented as ball and sticks and the interacting residues as sticks.

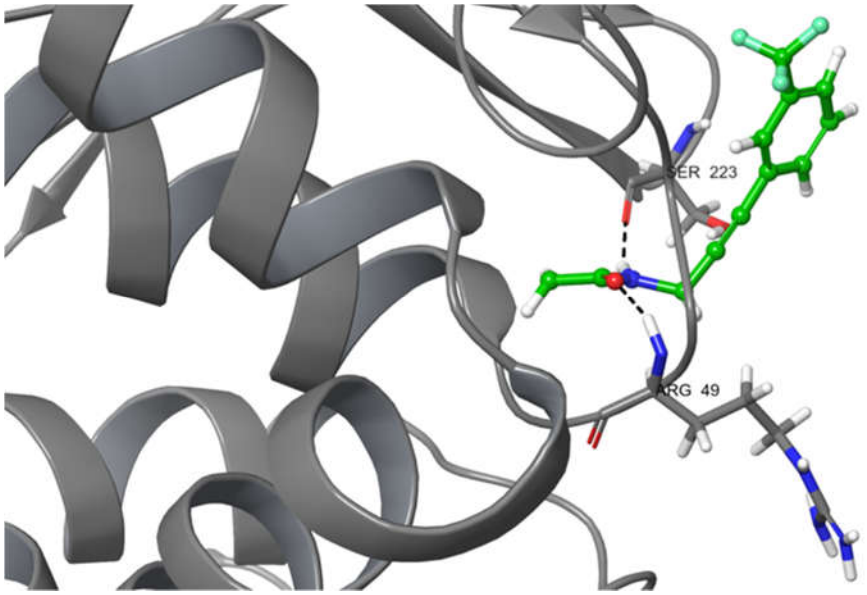

Figure S6. Dominating molecular interaction observed during the molecular dynamics simulation of the complex Otub-2 with compound PCM-0103011 (N-{3-[3-(trifluoromethyl)phenyl]prop-2-yn-1-yl}acetamide) - pdb id 5QIU. The covalent ligand is represented as ball and sticks and the interacting residues as sticks.

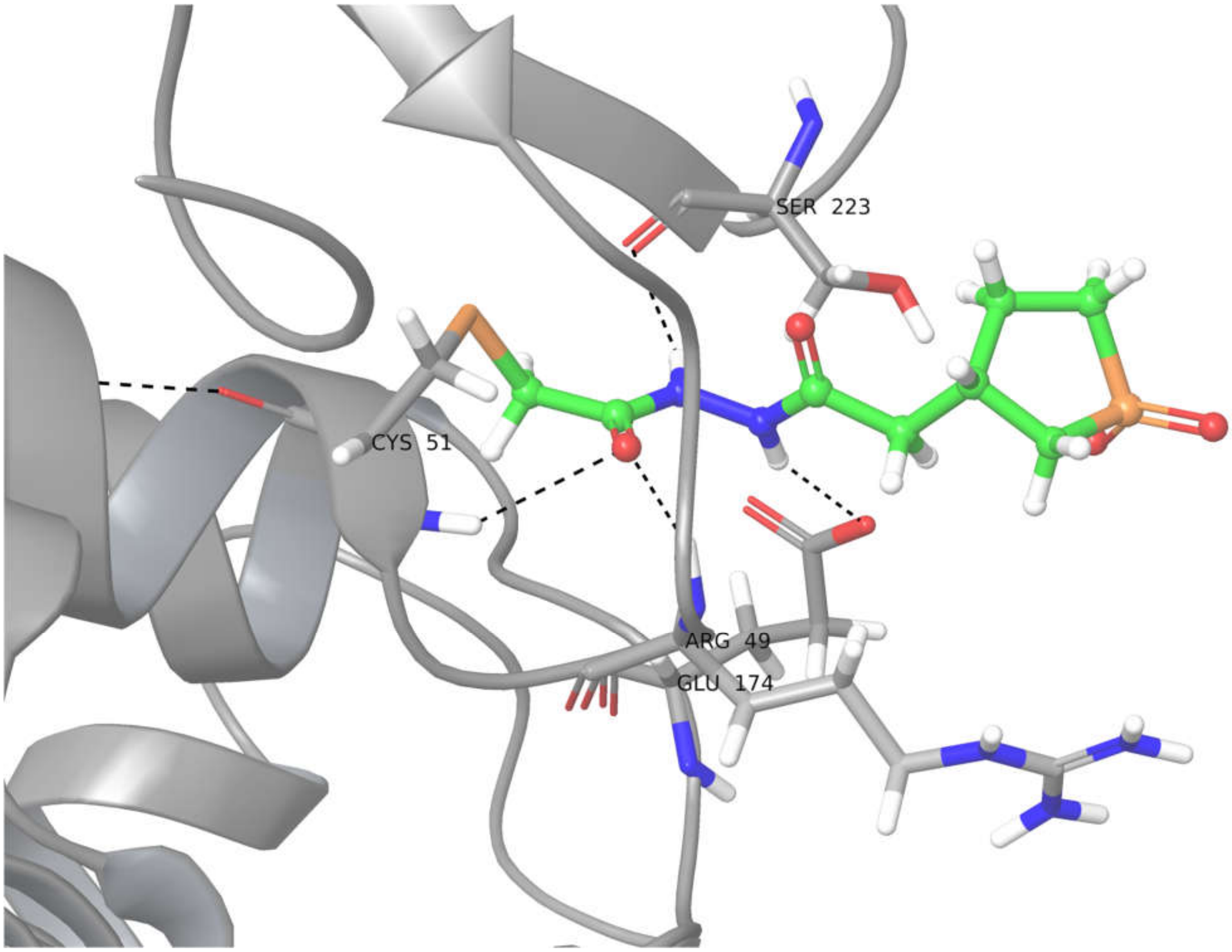

Figure S7. Dominating molecular interaction observed during the molecular dynamics simulation of the complex Otub-2 with compound PCM-0102998 (N'-acetyl-2-[(3R)-1,1-dioxo-1lambda~6~-thiolan-3-yl] acetohydrazide) - pdb id 5QIV. The covalent ligand is represented as ball and sticks and the interacting residues as sticks.

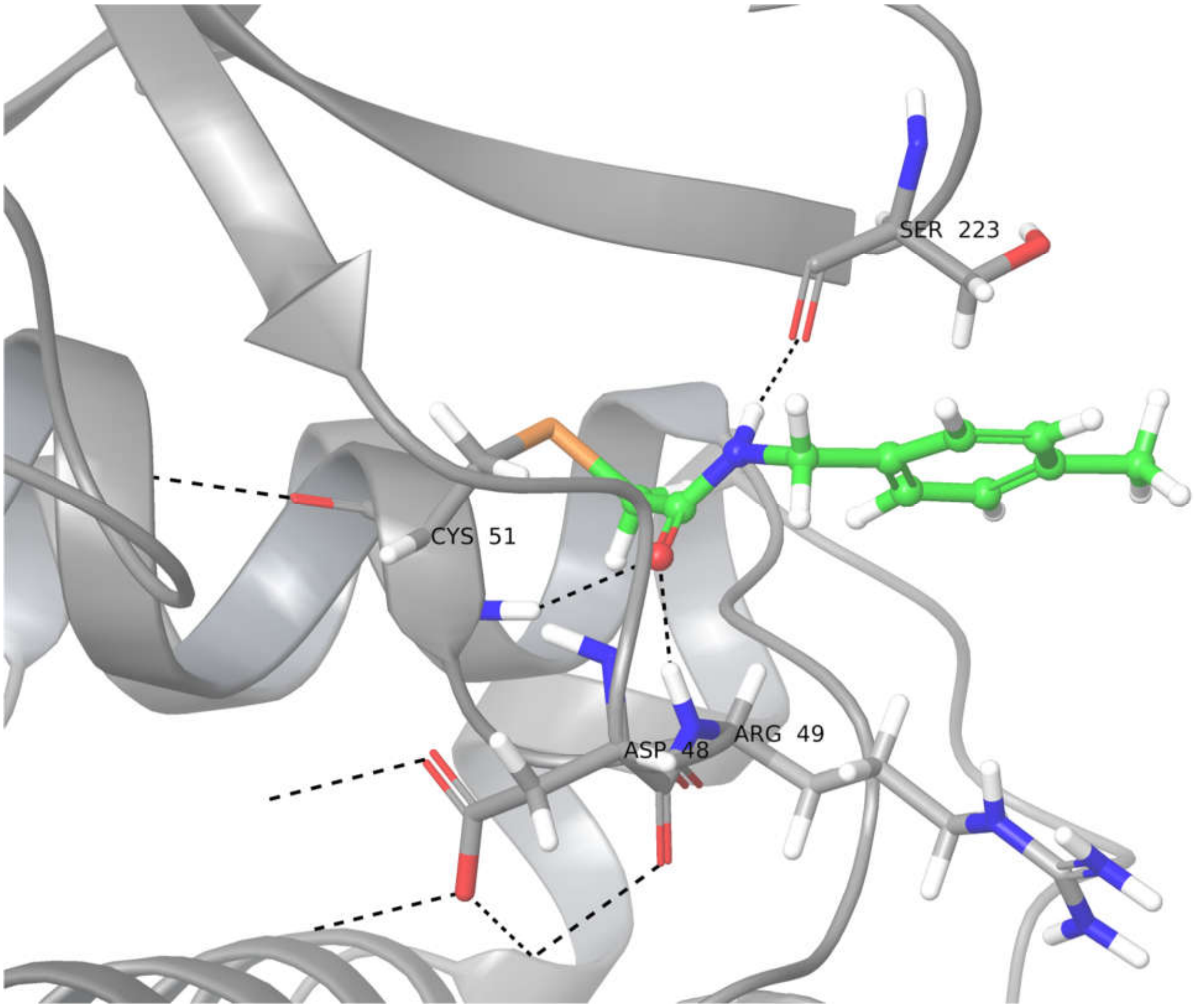

Figure S8. Dominating molecular interaction observed during the molecular dynamics simulation of the complex Otub-2 with compound PCM-0102660 (N-[(E)-(4-methylphenyl)methylidene] acetamide) - pdb id 5QIW. The covalent ligand is represented as ball and sticks and the interacting residues as sticks.

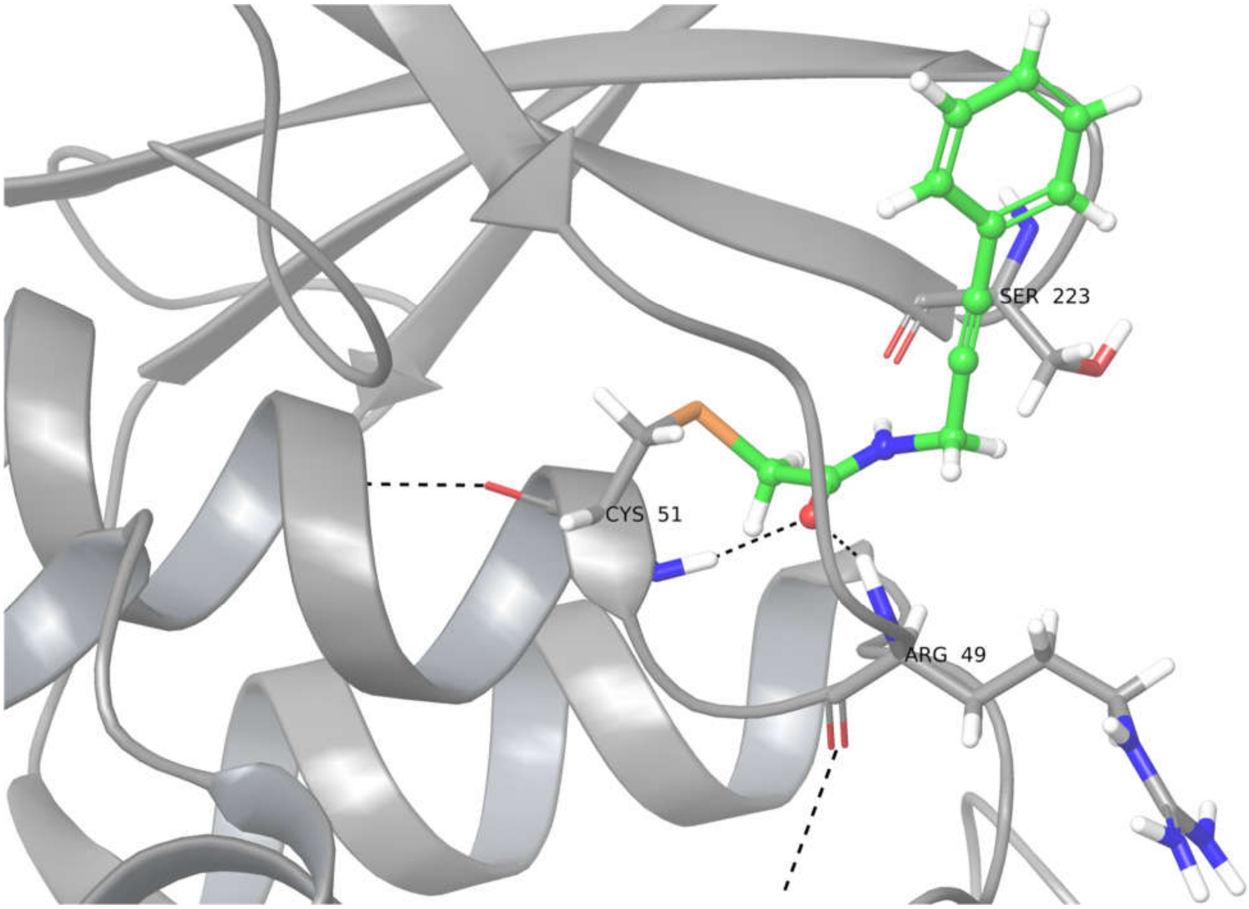

Figure S9. Dominating molecular interaction observed during the molecular dynamics simulation of the complex Otub-2 with compound PCM-0103007 (N-(3-phenylprop-2-yn-1-yl)acetamide) - pdb id 5QIX. The covalent ligand is represented as ball and sticks and the interacting residues as sticks.

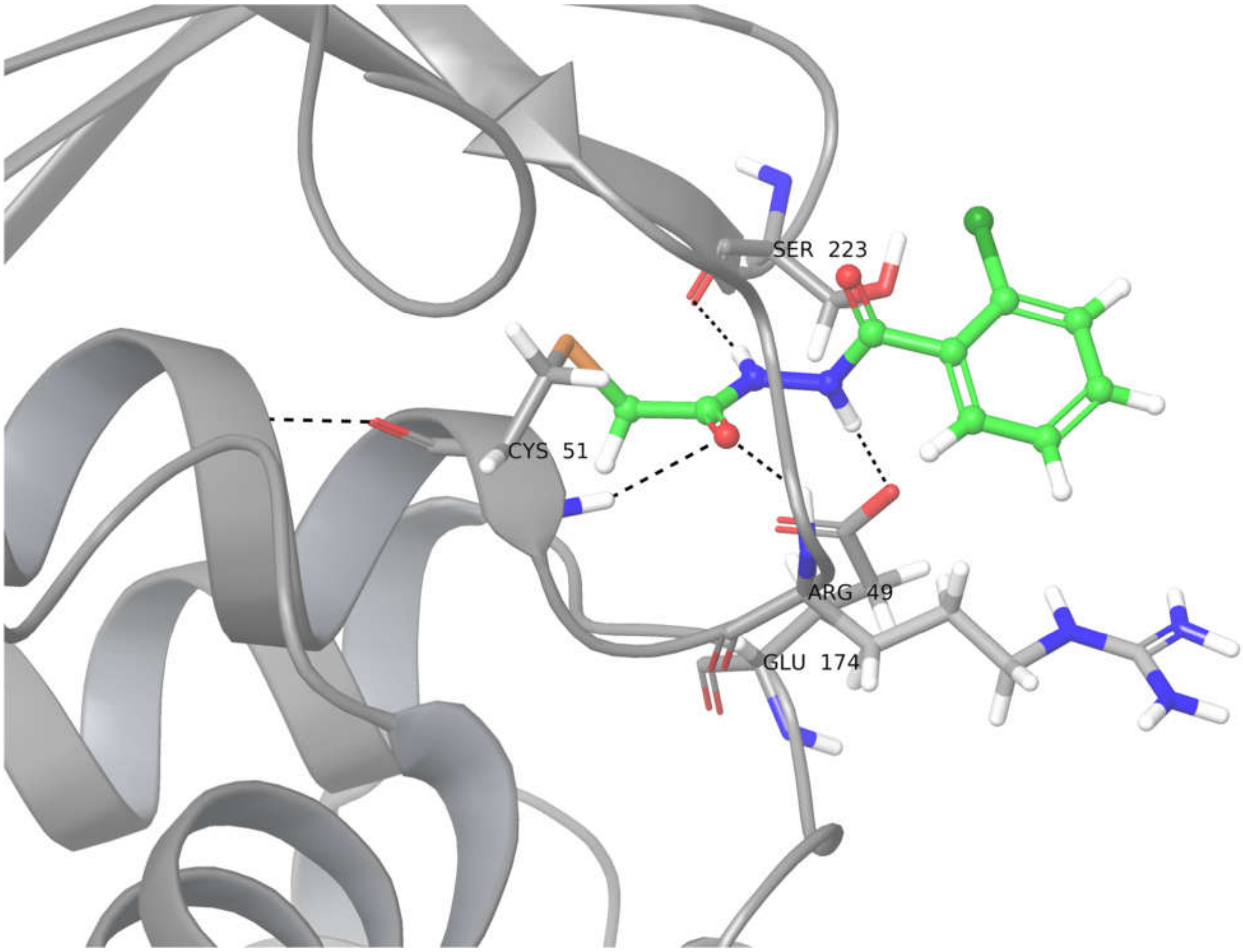

Figure S10. Dominating molecular interaction observed during the molecular dynamics simulation of the complex Otub-2 with compound PCM-0102954 (N'-acetyl-2-chlorobenzohydrazide) - pdb id 5QIY. The covalent ligand is represented as ball and sticks and the interacting residues as sticks.

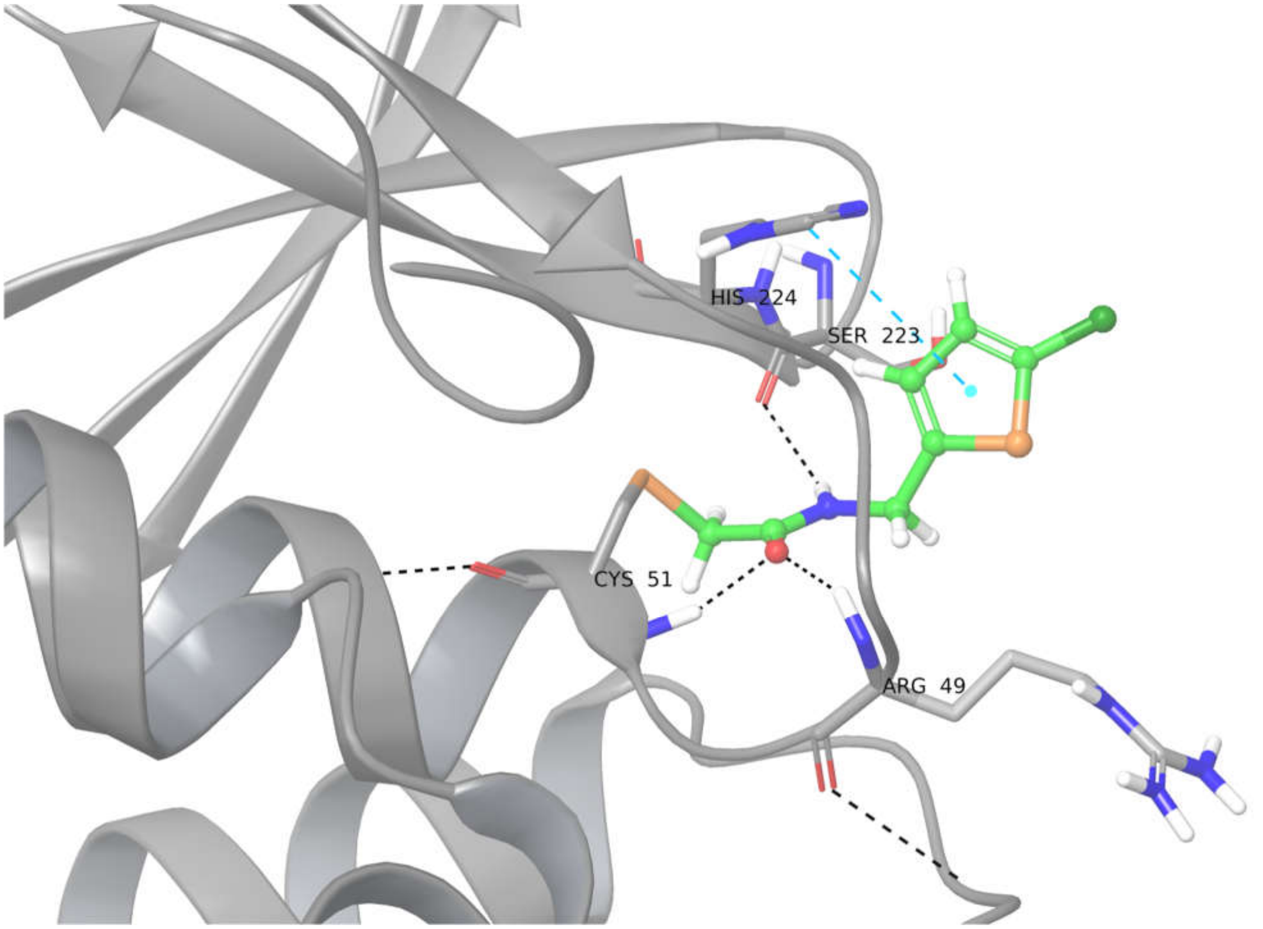

Figure S11. Dominating molecular interactions observed during the molecular dynamics simulation of the complex Otub-2 with compound PCM-0103080 (N-[(5-chlorothiophen-2-yl) methyl] acetamide) - pdb id 5QIZ. The covalent ligand is represented as ball and sticks and the interacting residues as sticks.

Table S1. Covalent selective inhibitors of the OTUB2 DUB. Comparison of co-crystallized ligand with MD refined structures.

PDB ID	Ligand 2D Structure	Ligand RMSD (Å)	Covalent Protein-OTUB2 Complex[a]
**5QIP**	**PCM-0102153** 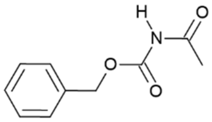	1.23±0.3	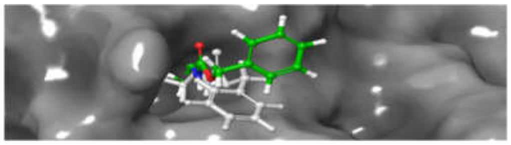
**5QIQ**	**PCM-0103050** 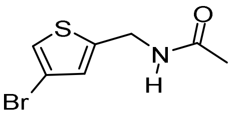	1.05±0.3	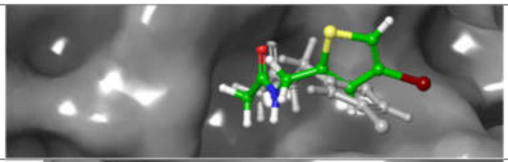
**5QIR**	**PCM-0102305** 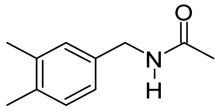	1.09±0.2	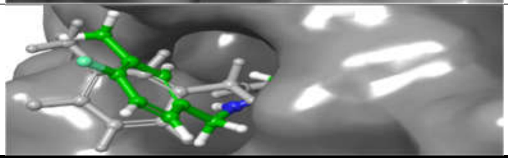

**5QIS**	**PCM-0102500** 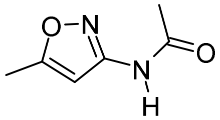	0.35±0.1	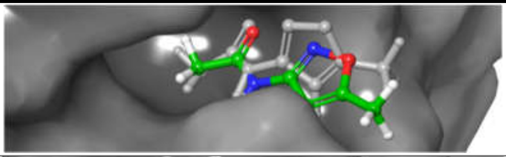
**5QIT**	**PCM-0102821** 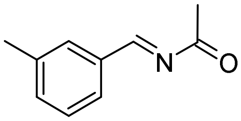	1.02±0.4	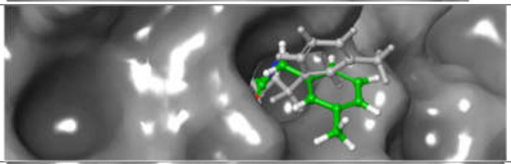
**5QIU**	**PCM-0103011** 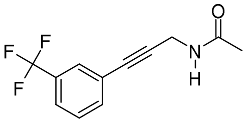	1.10±0.4	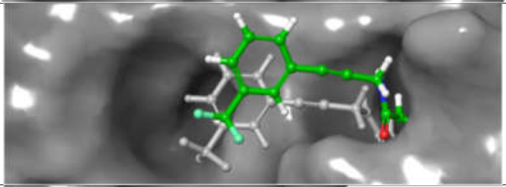
**5QIV**	**PCM-0102998** 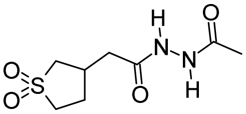	0.82±0.2	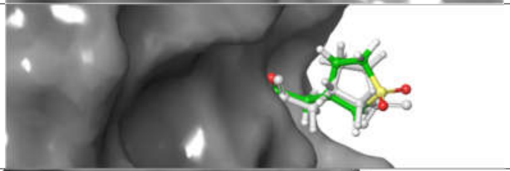
**5QIW**	**PCM-0102660** 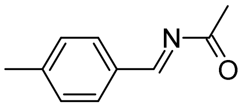	0.74±0.2	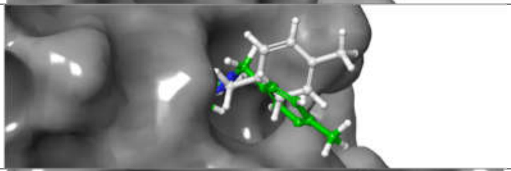
**5QIX**	**PCM-0103007** 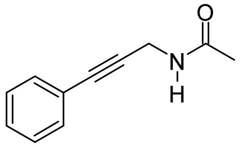	0.44±0.2	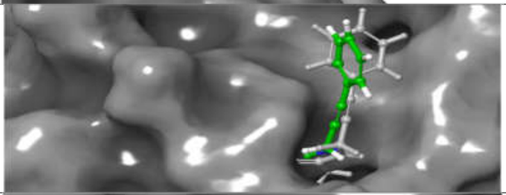
**5QIY**	**PCM-0102954** 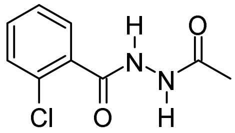	0.65±0.5	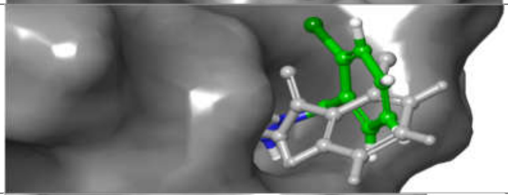
**5QIZ**	**PCM-0103080** 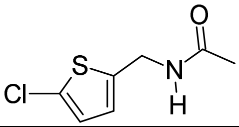	1.29±0.1	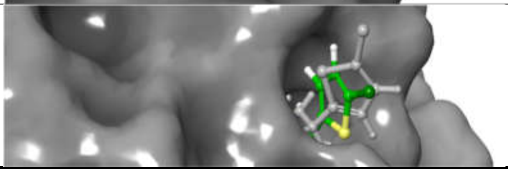

[a]Binding and orientation of covalently-bound OTUB2 inhibitors (grey: X-ray structure; color: MD refined)

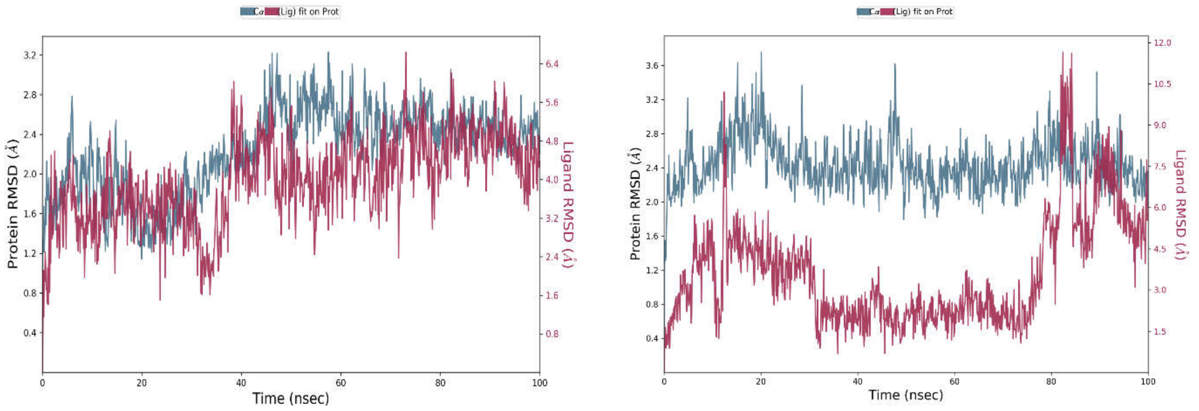

Figure S12. Molecular Dynamics studies of SARS-Cov-2 PlPro to show the RMSD of protein and Ligand over time for Compound 1 (left) and Compound 2 (right).

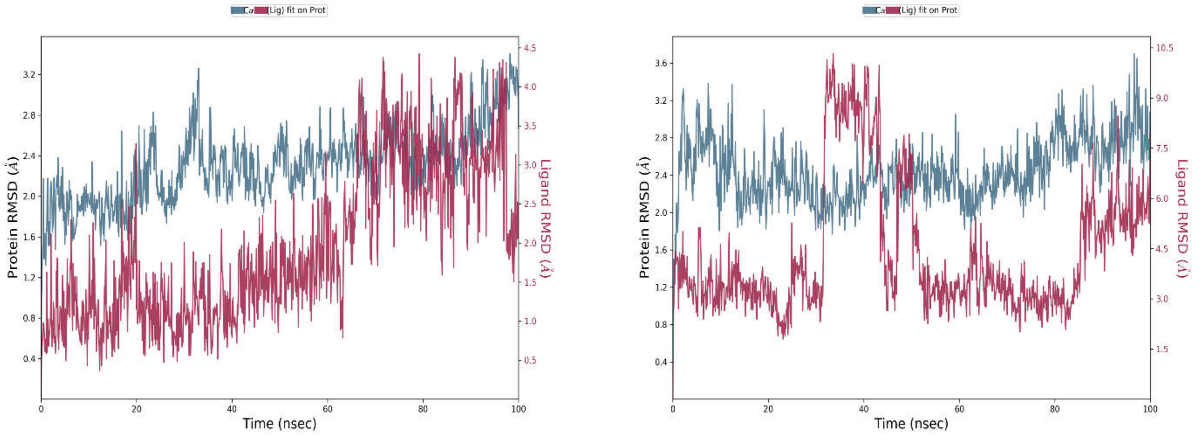

Figure S13. Molecular Dynamics studies of SARS-Cov-2 PlPro to show the RMSD of protein and Ligand over time for Compound 3 (left) and Compound 4 (right).

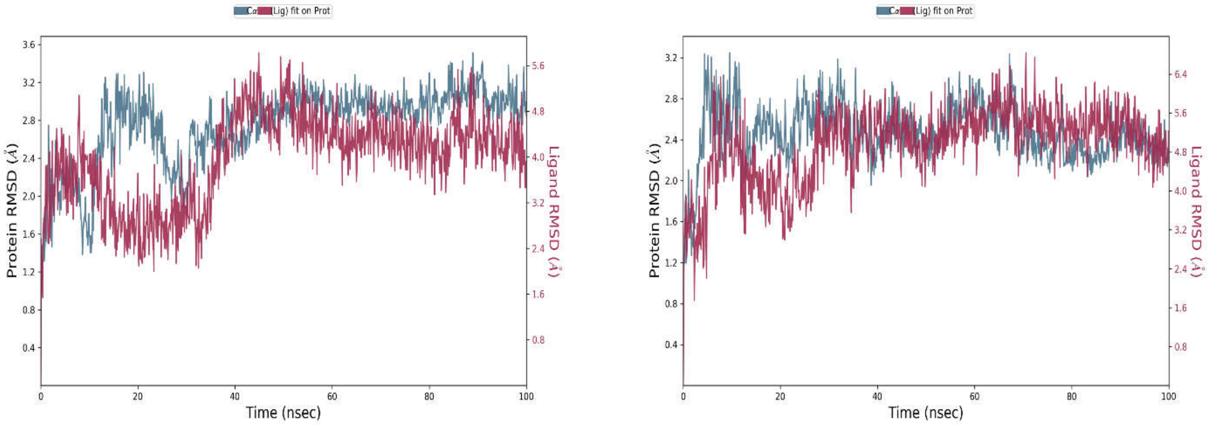

Figure S14. Molecular Dynamics studies of SARS-Cov-2 PlPro to show the RMSD of protein and Ligand over time for Compound 5 (left) and Compound 6 (right).

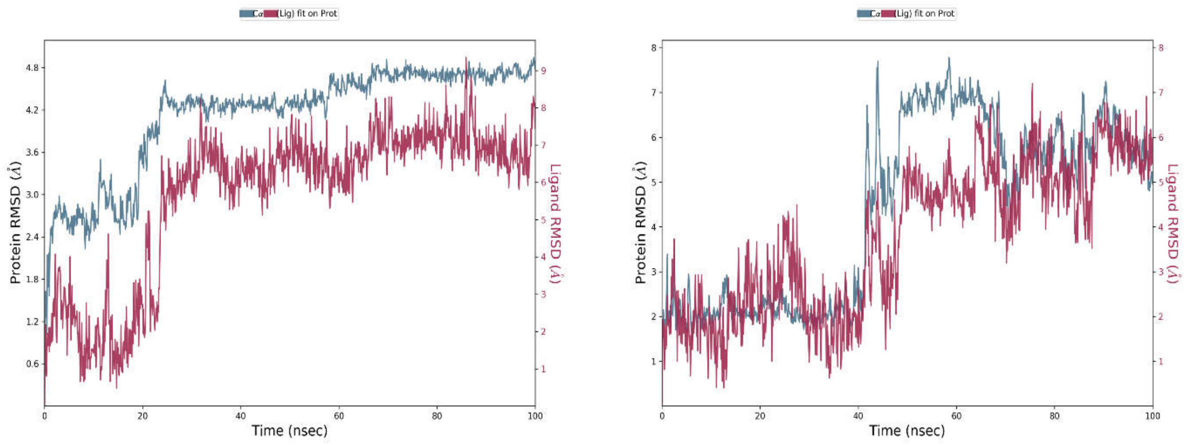

Figure S15. Molecular Dynamics studies of OTUB-1 to show the RMSD of protein and Ligand over time for Compound 1 (left) and Compound 2 (right).

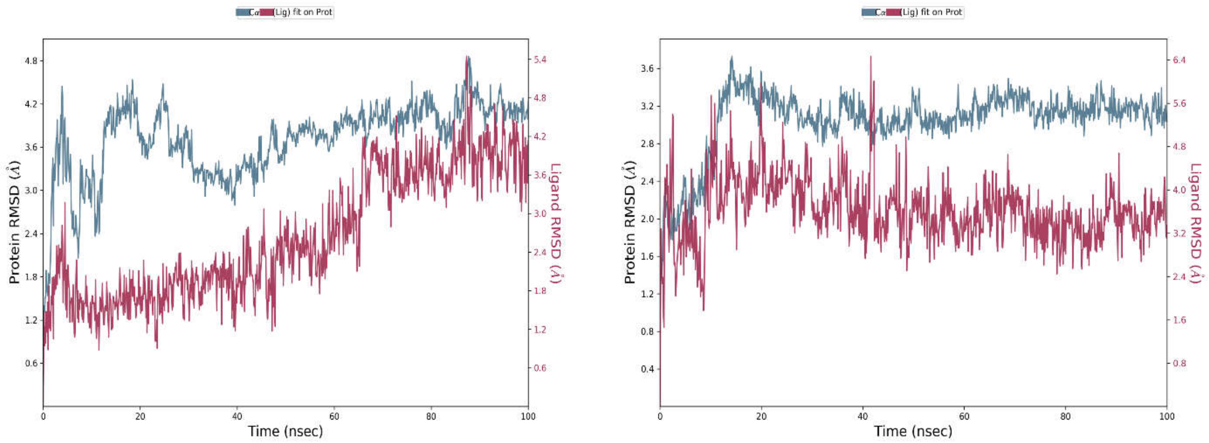

Figure S16. Molecular Dynamics studies of OTUB-1 to show the RMSD of protein and Ligand over time for Compound 3 (left) and Compound 4 (right).

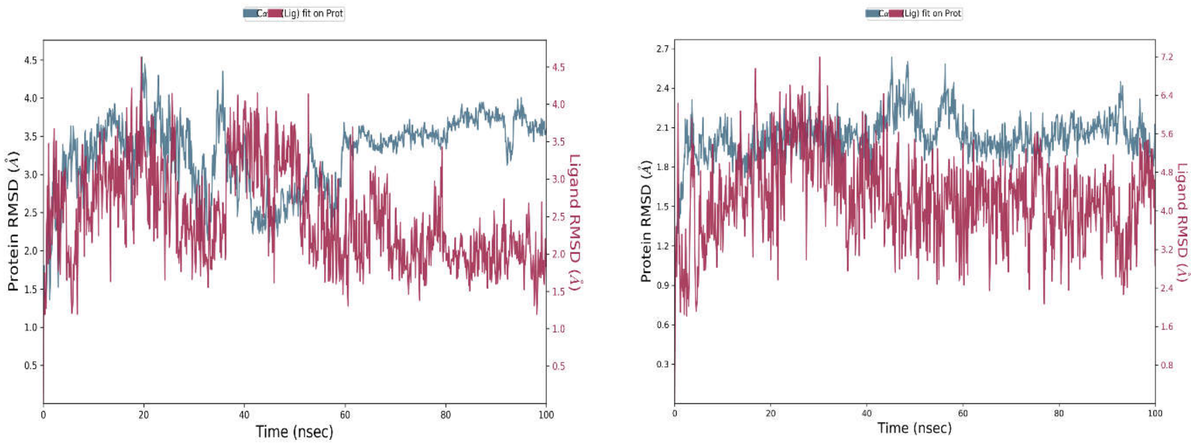

Figure S17. Molecular Dynamics studies of OTUB-1 to show the RMSD of protein and Ligand over time for Compound 5 (left) and Compound 6 (right).

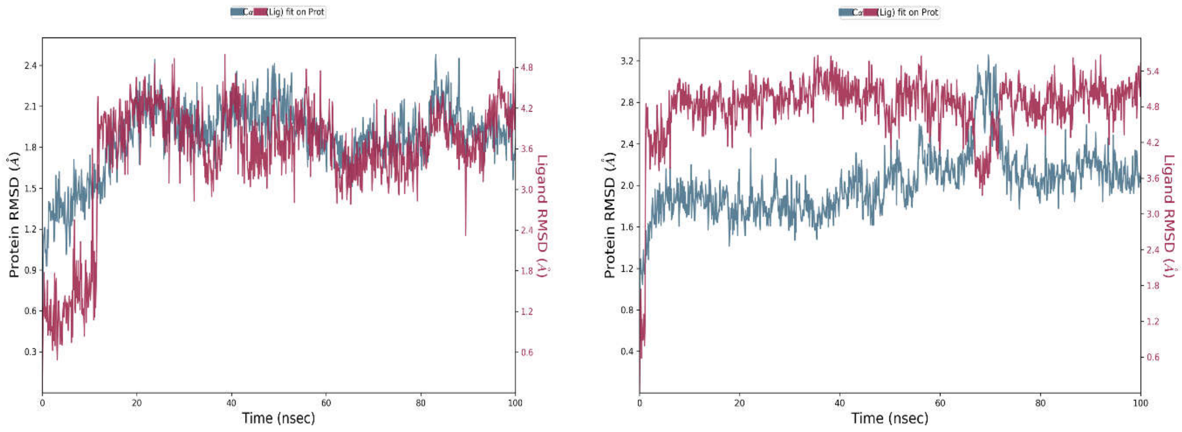

Figure S18. Molecular Dynamics studies of OTUB-2 to show the RMSD of protein and Ligand over time for Compound 1 (left) and Compound 2 (right).

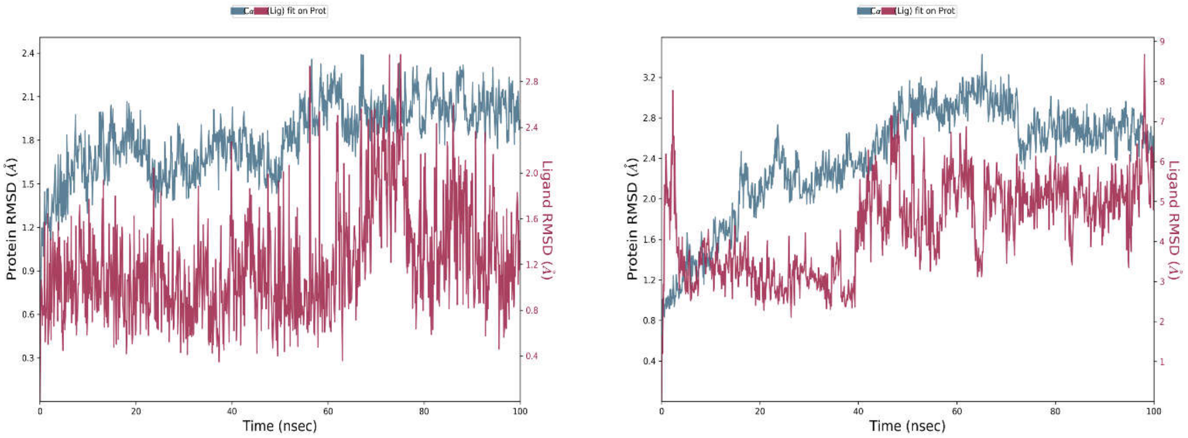

Figure S19. Molecular Dynamics studies of OTUB-2 to show the RMSD of protein and Ligand over time for Compound 3 (left) and Compound 4 (right).

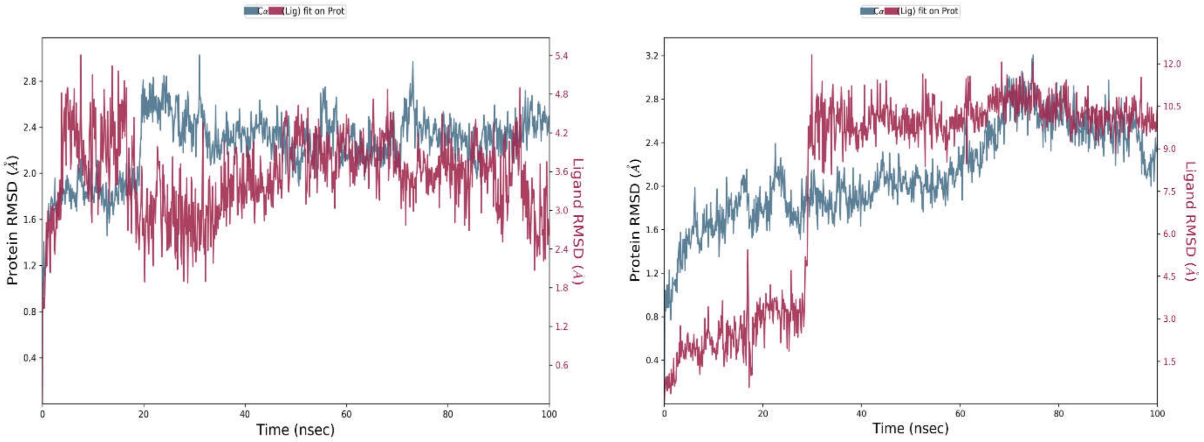

Figure S20. Molecular Dynamics studies of OTUB-2 to show the RMSD of protein and Ligand over time for Compound 5 (left) and Compound 6 (right).

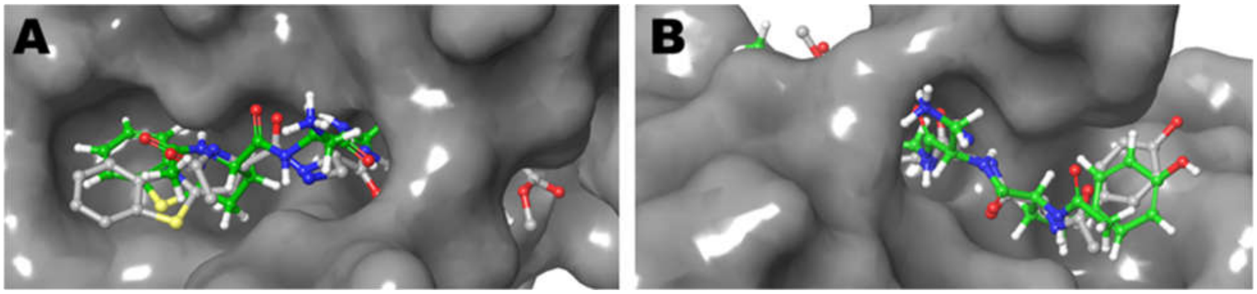

Figure S21. Cov-2-PLpro with its inhibitors in the X-ray structures (ligand carbon in grey) and refined with MD simulations (ligand carbon in green). A) 6WUU with peptide inhibitor **VIR250** and B) 6WX4 with peptide inhibitor **VIR251**.

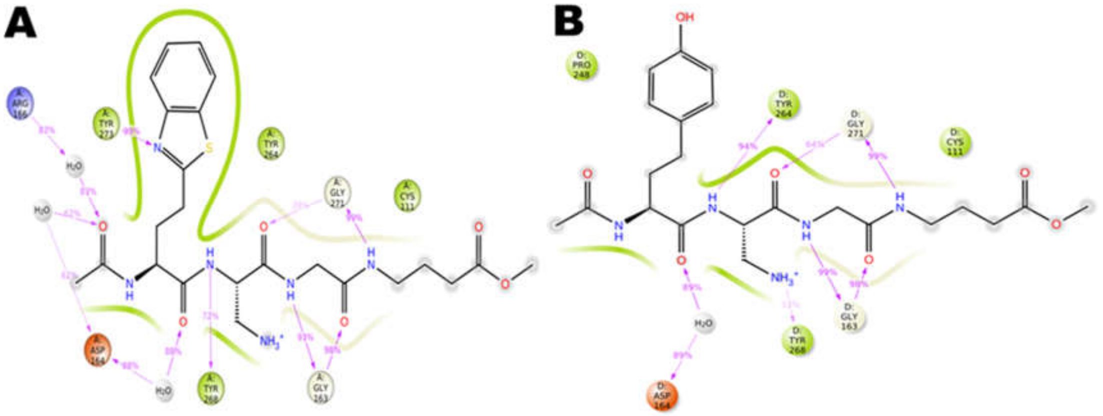

Figure S22. Dominating non-covalent protein-ligand interactions of the peptide inhibitors A) **VIR250**, B) **VIR251** with the PLpro SARS-CoV-2 during MD refinement simulations. Persistence of interactions is given in percent of simulation time.
